# Trends in acupuncture for infertility: a scoping review with bibliometric and visual analysis

**DOI:** 10.3389/fendo.2024.1351281

**Published:** 2024-06-04

**Authors:** Ziyu Tian, Chongyang Zhang, Xing Liao, Sihong Yang, Yuying Hong, Anni Shi, Fei Yan, Ting Pan, Jiajia Zhang, Yan Meng, Nicola Robinson, Peng Bai, Weijuan Gang

**Affiliations:** ^1^ Dongzhimen Hospital, Beijing University of Chinese Medicine, Beijing, China; ^2^ Institute of Acupuncture and Moxibustion, China Academy of Chinese Medical Sciences, Beijing, China; ^3^ Third Affiliated Hospital, Beijing University of Chinese Medicine, Beijing, China; ^4^ Center for Evidence Based Chinese Medicine, Institute of Basic Research in Clinical Medicine, China Academy of Chinese Medical Sciences, Beijing, China; ^5^ China Center for Evidence Based Traditional Chinese Medicine, China Academy of Chinese Medical Sciences, Beijing, China; ^6^ School of Acupuncture-Moxibustion and Tuina, Beijing University of Chinese Medicine, Beijing, China; ^7^ Department of Gynecology, Dongzhimen Hospital, Beijing University of Chinese Medicine, Beijing, China; ^8^ Department of Acupuncture and Moxibustion, Beijing Longfu Hospital, Beijing, China; ^9^ Centre for Evidence-Based Chinese Medicine, Beijing University of Chinese Medicine, Beijing, China; ^10^ Institute of Health and Social Care, London South Bank University, London, United Kingdom

**Keywords:** infertility, natural conception, acupuncture, scoping review, bibliometric and visual analysis

## Abstract

**Background:**

Unexplained recurrent implantation failure and the high cost of assisted reproductive techniques for those experiencing infertility have increasingly resulted in the use of acupuncture. However, the trends and research status of acupuncture on infertility resulting in natural conception have not been systematically summarized. This scoping review and knowledge graph analysis aimed to summarize existing clinical studies on acupuncture for infertility that resulted in natural conception.

**Methods:**

Seven databases, namely, PubMed, Embase, the Cochrane Library, CNKI, VIP, Wanfang Data, and SinoMed, were searched up to August 2023 (updated on 1 April). Two authors independently identified related clinical studies and systematic reviews, and extracted data from included studies on acupuncture for infertility; any discrepancies were resolved by discussion or judged by a third author. A meta-analysis was conducted based on randomized controlled trials (RCTs), and data were synthesized using risk ratios with 95% confidence intervals.

**Results:**

Of the 310 articles meeting the inclusion criteria, 274 were primary studies, 7 were systematic reviews, and 29 were case reports. Reported adverse events included mild ovarian irritation and early signs of miscarriage. Out of the 274 primary studies, there were 40 (14.60%) cases of male infertility and 234 (85.40%) cases of female infertility. Current research highlights on acupuncture for infertility focused on female infertility caused by polycystic ovary syndrome, ovulation disorder, and luteinized unruptured follicle syndrome (LUFS), while acupuncture for male infertility was a hotspot in the early research stage. The meta-analysis also suggested that acupuncture was more effective than human chorionic gonadotropin (HCG) [RR = 1.89, 95% CI (1.47, 2.42), 11 RCTs, 662 participants]. Acupuncture combined with HCG was comparable to HCG [RR = 2.33, 95% CI (1.53, 3.55), four RCTs, 259 participants]. Compared with no treatment, acupuncture resulted in a higher pregnancy rate [RR = 22.12, 95% CI (1.39, 353.09), one RCT, 47 participants]. There was no statistical difference between acupuncture combined with HCG plus letrozole and HCG plus letrozole [RR = 1.56, 95% CI (0.84, 2.89), one RCT, 84 participants].

**Conclusion:**

Current research highlights on acupuncture for infertility resulting in natural conception focused on female infertility caused by polycystic ovary syndrome, ovulation disorder, and LUFS, while studies on male infertility and female infertility caused by blockage in the fallopian tube, thin endometrium, and other factors were insufficient. Well-designed confirmatory clinical studies are still needed as the research hypotheses of most studies were unclear.

## 1 Introduction

Infertility is defined as the failure to become pregnant within 1 year of regular and unprotected intercourse, which may be related to a number of factors or unexplained reasons (1). As the problem may lead to a series of psychological distress, social stigmatization, economic strain, and even marital discord, it has been considered as a public health priority (2). Globally, the disability-adjusted life-years (DALYs) and the global disease burden of infertility also increased for both women and men throughout 1990 to 2017, and the age-standardized prevalence infertility for women and men increased by 14.96% and 8.22%, respectively, for this period (3). It has affected at least 180 million reproductive-aged couples worldwide (4).

In general, the prevalence of infertility among women is higher than among men; for example, in UK, the estimated prevalence of infertility was 12.5% for women and 10.1% for men (5). Recent research suggests that men are solely responsible for 20%–30% of infertility cases (4, 6). Female infertility can be caused by diseases such as pelvic lesions and ovulation disorders, while male infertility can also be caused by various diseases or factors, such as dysspermia and male sexual dysfunction (4). Conventional therapy for infertility involves drug treatment (clomiphene and gonadotropin-releasing hormone analogs) and assisted reproductive techniques (ARTs), such as *in vitro* fertilization (IVF), hormonal stimulation, and intracytoplasmic sperm injection (ICSI) (7), which can overcome male and female infertility. Even uterus transplantation has been proposed as a potential choice for uterine infertility, but it also brings further ethical challenges such as the selection of the donor, the impact on the recipient and offspring, and ethical and social challenges (8). The efficacy of a sole drug can be limited, although ART has advanced the outcomes of conception for these couples, and the live birth rates, through IVF or ICSI, are between 30% and 35% (9, 10). These technologies require strict conditions during their delivery, but can cause serious health problems including antepartum hemorrhage, congenital anomalies, preterm rupture of the membranes, low birth weight, perinatal mortality, preterm delivery, and gestational diabetes. Only 20% and 35% on initiated cycles and embryo transfer cycles result in the birth of a healthy baby, respectively (11–13). The adverse effects of ART in most countries, especially in low-income and middle-income countries, and the availability, accessibility, and quality of such infertility interventions remain a major challenge due to the lack of trained personnel and the necessary equipment and infrastructure (14).

Acupuncture has a long history for the treatment of female and male reproductive disease in China. Basic research has showed that acupuncture can affect gonadotropin-releasing hormone secretion and the menstrual cycle, and improve the blood flow of uterus (15). Previous systematic reviews have shown that acupuncture can decrease the rate of pregnancy loss (16), and there are also studies that have shown that acupuncture can improve fertility outcomes and mental health in both men and women (17, 18). Owing to a series of adverse events, unexplained recurrent implantation failure, or the high cost of ART, more patients tend to choose acupuncture as a conservative treatment (17). However, the benefits of acupuncture alone or combined with Western therapy on infertility have not been systematically summarized.

A scoping review aims to map types of evidence and identify current research gaps of an exploratory clinical research question (19). There is no comprehensive summary of acupuncture for infertility that resulted in natural conception; thus, this scoping review aimed to summarize existing clinical studies on acupuncture for infertility that resulted in natural conception among the population, the acupuncture method (including source of acupuncture, frequency, and course), and the consistency between research hypotheses and conclusions.

## 2 Methods

This study was conducted according to the Preferred Reporting Items for Systematic Reviews and Meta-Analyses (PRISMA) guidelines extended edition for scoping reviews (20).

### 2.1 Eligibility criteria

#### 2.1.1 Types of studies

Randomized controlled trials (RCTs), non-RCTs, cohort studies, case–control studies, case series studies, case reports, and secondary studies (such as systematic reviews and/or meta-analyses, overview of systematic reviews, expert consensus or clinical guidelines) were included in this review without limitation on language, date, and form of publication.

#### 2.1.2 Types of participants

Individuals experiencing problems with infertility caused by either female factors such as pelvic lesions and ovulation disorders or male factors such as dysspermia, male sexual dysfunction, and unexplained infertility were included.

#### 2.1.3 Types of interventions

The intervention group was defined as acupuncture therapy alone or acupuncture plus conventional drugs (such as ovulation induction medications and hormone therapy medications) or surgeries (such as laparoscopic surgery and varicocelectomy).

The exclusion criteria were as follows: (1) acupuncture combined with Chinese herbal medicines whether in the intervention group or the control group; (2) the control group receiving acupuncture; and (3) undergoing ART treatment (including artificial insemination, embryo transfer, IVF, ISCI, pre-implantation genetic diagnosis, embryo freezing and frozen embryo transfer technology, and *in vitro* maturation technology).

#### 2.1.4 Types of outcomes

This study mainly focused on acupuncture for natural conception. Outcomes were pregnancy rate and live birth rate for women, and pregnancy rate (their partner) and semen parameters (including concentration, total number, vitality, and normal morphology) for men.

### 2.2 Search strategies

A systematic search of electronic databases was performed, including PubMed, Cochrane Central Register of Controlled Trials (CENTRAL; The Cochrane Library), Cochrane Database of Systematic Reviews (CDSR; The Cochrane Library), EMBASE, China Network Knowledge Infrastructure (CNKI), China Science and Technology Journal Database, Wanfang Data, and SinoMed (searched from onset until 5 August 2023, updated on 1 April 2024); there were no language and publication restrictions. The search strategy on PubMed is given in **Additional File 1**: **Supplementary Table S1**.

### 2.3 Data collection and analysis

#### 2.3.1 Selection of studies

The studies were exported to EndNote software (V20) for management. Duplicate studies were removed independently by CYZ. The remaining studies were screened independently by TP and JJZ according to the inclusion/exclusion criteria. Firstly, the two researchers excluded unrelated studies by reading their titles and abstracts, then they acquired the full text for further screening. Any discrepancies were resolved by discussion or judged by a third author.

### 2.4 Bibliometric analysis and data extraction

The included literature was exported into CiteSpace 6.3.R1 software to convert the data format, then a collaborative network analysis on authors and institutions from literature, co-occurrence network analysis, and citation burst on keywords were conducted. The years covered were from 1994 to 2024, with a time slice of 2 years and a cutting method including pathfinder, pruning sliced networks, and pruning the merged network.

As for the characteristics of the included literature, the list was set up by CYZ using Microsoft Excel 2007. Data extraction was completed after cross-checking by YYH and ANS; FY and YM extracted the data independently. The following data were extracted according to PRISMA and the PICO (patient, intervention, comparison and outcome) framework: first author’s name, publication year, title, journal, the type of literature, language, funding, design of research, objective, patients (sample size), disease/pathogenesis, the treatment of the control group, acupoint prescriptions and their rationale for selection, frequency and duration of acupuncture treatment, adverse events, follow-up, research hypotheses, outcomes, and main conclusions.

Descriptive analysis of data was carried out by calculating frequency and percentage, which was presented by tables and charts set up in Microsoft Excel 2007.

### 2.5 Quality assessment and data synthesis

When conducting meta-analysis on included RCTs, two reviewers (CYZ and SHY) independently assessed the quality of RCTs by using the Cochrane Risk of Bias tool (ROB), which included random sequence generation, allocation concealment, blinding of participants and personnel, blinding of outcome assessment, incomplete outcome data, selective reporting, and publication bias. Any discrepancies were resolved by discussion or judged by a third author.

Meta-analysis was performed using Review Manager 5.3. Risk ratio (RR) with 95% confidence interval (CI) was used to measure dichotomous variables, and mean difference (MD) or standardized mean difference (SMD) was used to measure continuous variables. When results were measured on the same scale, the outcomes were reported as MD; otherwise, the results were reported as SMD. Heterogeneity test was performed by chi-square test and presented as the value of *I* ^2^ statistics. When *I* ^2^ ≤ 50% and *p* ≥ 0.10, the fixed-effects model was used; otherwise, the random-effects model was used. The subgroup analysis was conducted to explore any factors that might explain the heterogeneity; if there was severe heterogeneity, sensitivity analysis was conducted to explore the potential sources of heterogeneity. Overall effects with a *p*-value below 0.05 were considered statistically significant.

### 2.6 Publication bias

A funnel plot was used to assess potential publication bias in a single meta-analysis involving 10 or more trials.

## 3 Results

### 3.1 Description of studies

**Figure 1:**
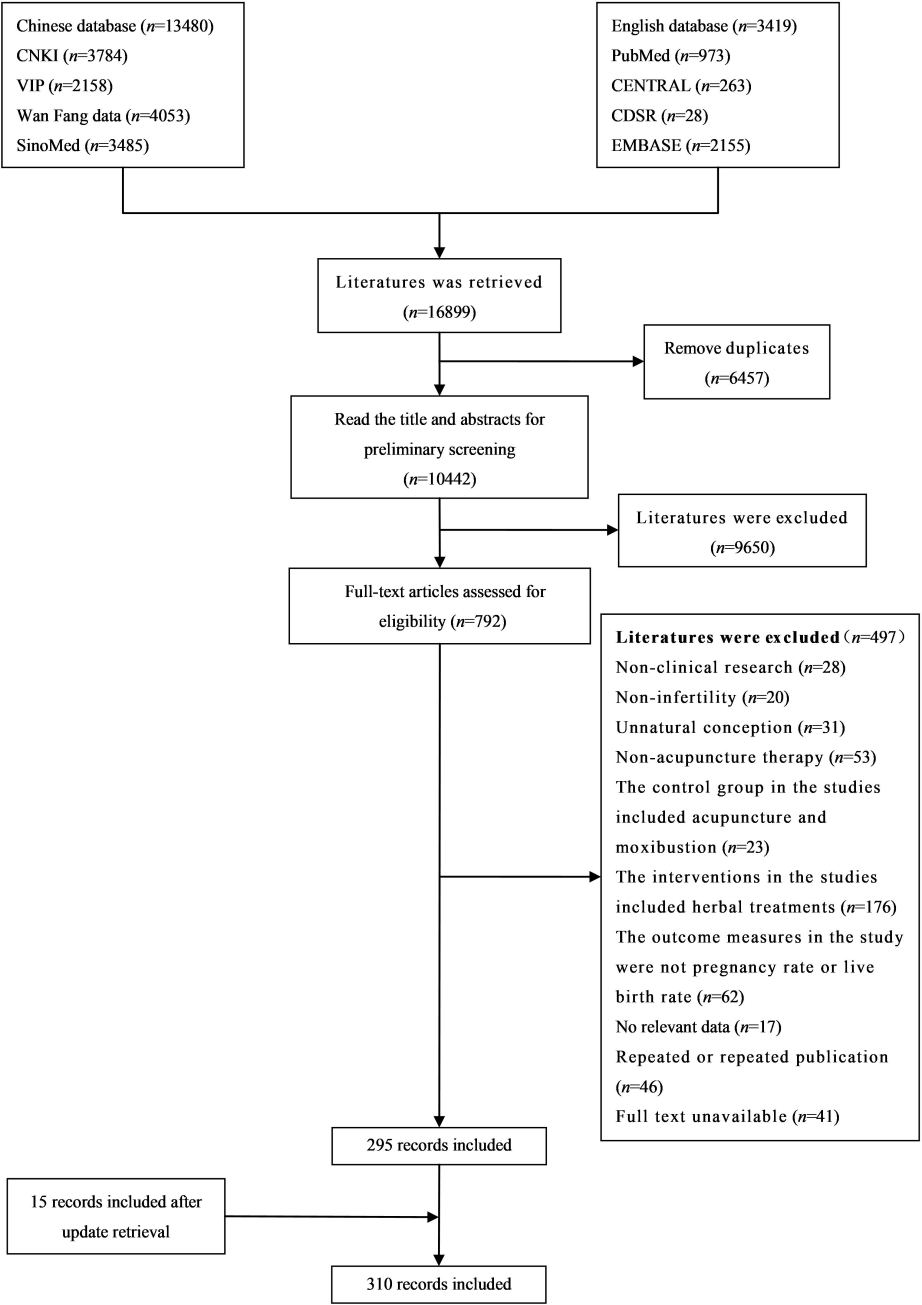
Flowchart of study selection.

Of the total of 16,899 articles identified in the search, 10,442 remained after duplicates were removed. A total of 9,650 articles were subsequently removed after screening by title and abstract. After full-text screening, 295 articles were included, and after supplementary searching on 1 April 2024, 15 articles were added; thus, there were 310 articles included. Data were extracted from 274 original studies and 7 meta-analyses. The remaining 29 case reports were just identified, but data were not extracted. For the study selection results, see **Figure 1**.

#### 3.1.1 Basic information of included studies

**Figure 2:**
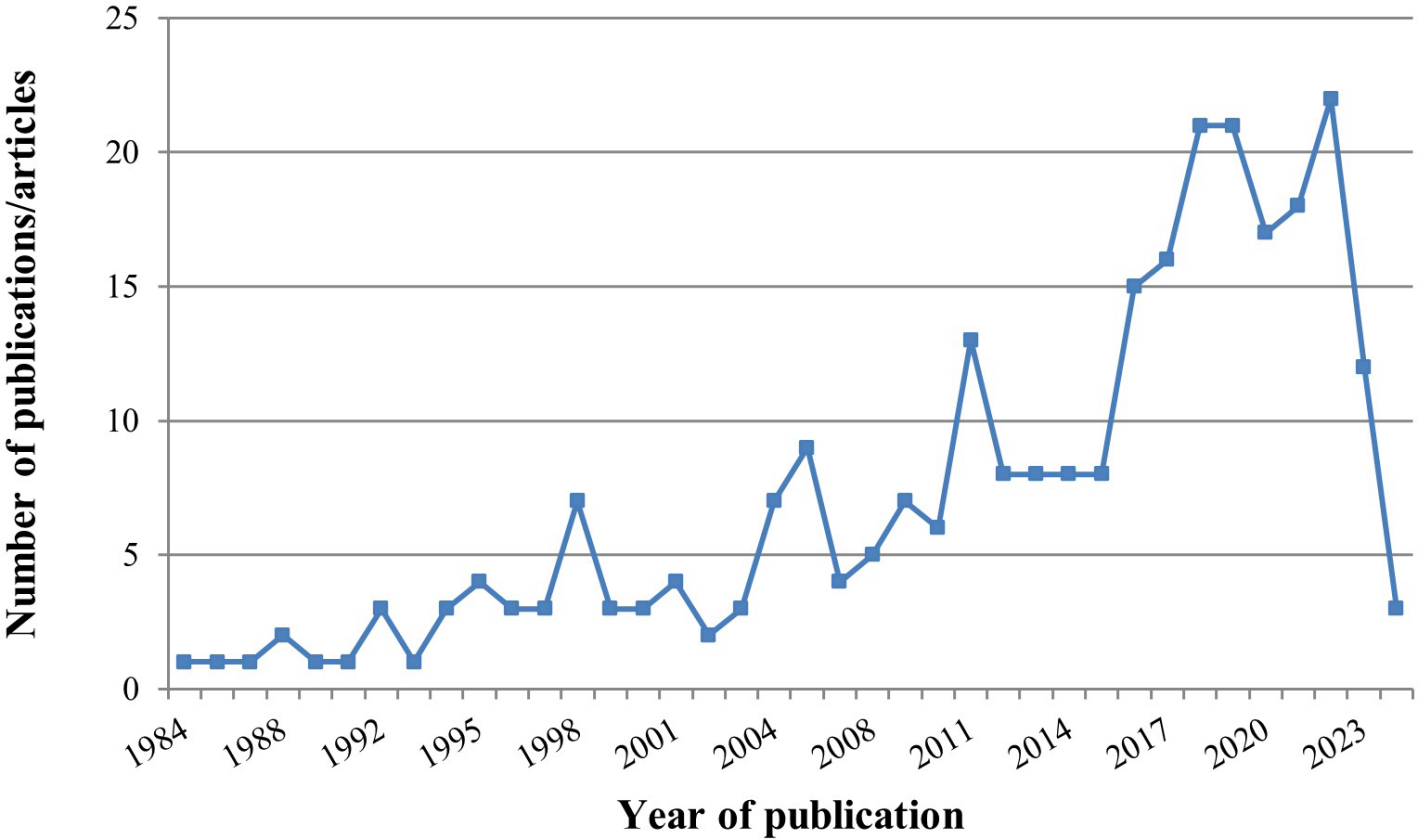
Chart of annual publications on acupuncture treatment for infertility.

The 274 studies included 192 (70.07%) RCTs, 73 (26.64%) case series, 5 (1.82%) non-RCTs, 3 (1.09%) cohort studies, and 1 (0.36%) matched controlled study (the characteristics of the extracted 274 studies are given in **Additional File 1**: **Supplementary Tables S2, S3**). Among them, 263 studies were in Chinese (95.99%) and 11 studies were in English (4.01%). The 274 studies consisted of 238 journal papers (86.86%), 34 master’s theses (12.41%), and 2 conference papers (0.73%). The 238 journal articles were published in 114 different journals. There was an increasing overall trend in the number of publications year on year (seen in **Figure 2**).

#### 3.1.2 Authors and institutions

**Figure 3:**
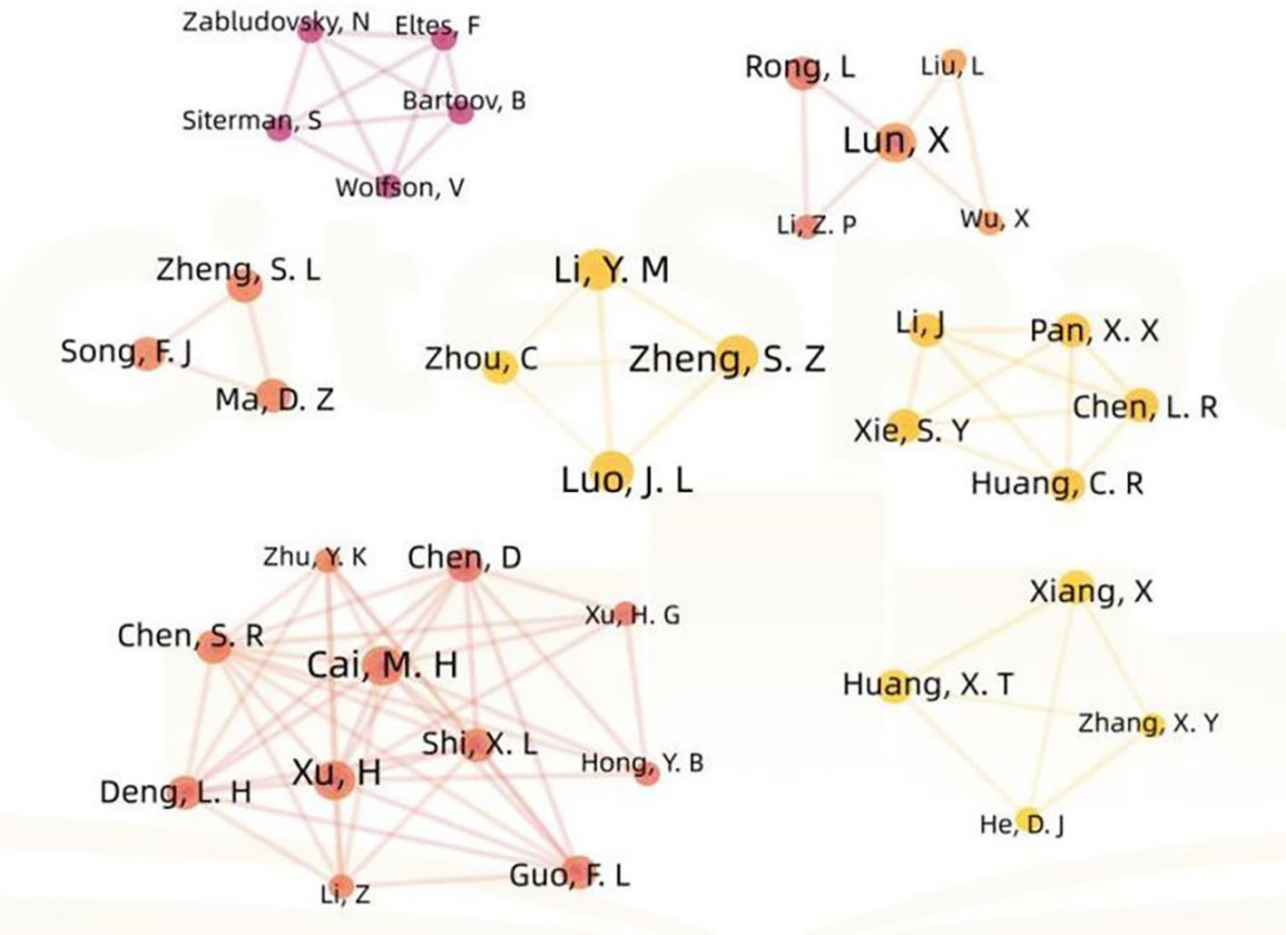
Cooperation network of authors in the field of acupuncture for infertility.

**Figure 4:**
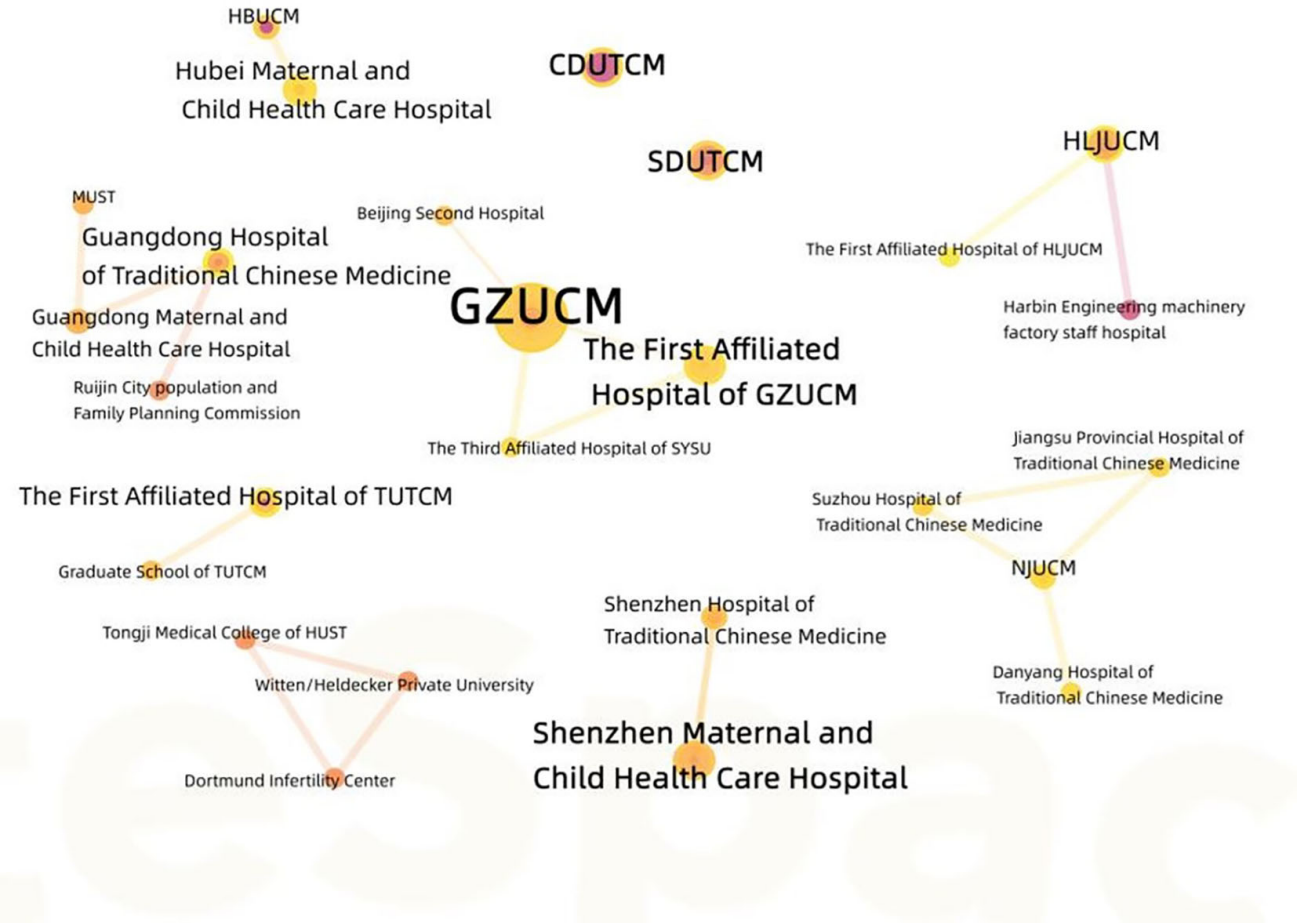
Cooperation network of institutions in the field of acupuncture for infertility.

There are a total of six authors who published three articles, while the other authors published less than three articles. The authors are all relatively stable but dispersed in small-scale cooperative networks, the contribution rate of different authors to the discipline varies depending on their publication volume, the core authors with higher contribution rates gather into a core author group, and no core author group has yet been formed in this field (see **Figure 3**). The top 10 institutions with the highest number of publications are shown in **Table 1**. Considering different institutions as nodes, nodes with a centrality exceeding 0.1 are referred to as key nodes, and due to the fact that the centrality of different institutions has not reached the target value, a cooperative network between different institutions has not been formed (see **Figure 4**).

**Table 1:** Top 10 productive institutions in the field of acupuncture for infertility.

Number	Institution	Number of publications/articles
1	GZUCM	18
2	HLJUCM	5
3	SDUTCM	4
4	CDUTCM	4
5	The First Affiliated Hospital of GZUCM	4
6	Shenzhen Maternal and Child Health Care Hospital	4
7	FJTCM	3
8	Hubei Maternal and Child Health Care Hospital	3
9	The First Affiliated Hospital of TUTCM	3
10	Guangdong Hospital of Traditional Chinese Medicine	3

GZUCM, Guangzhou University of Chinese Medicine; HLJUCM, Heilongjiang University of Chinese Medicine; SDUTCM, Shandong University of Traditional Chinese Medicine; CDUTCM, Chengdu University of Traditional Chinese Medicine; FJTCM, Fujian University of Traditional Chinese Medicine; TUTCM, Tianjin University of Traditional Chinese Medicine.

### 3.2 Keyword analysis

**Figure 5:**
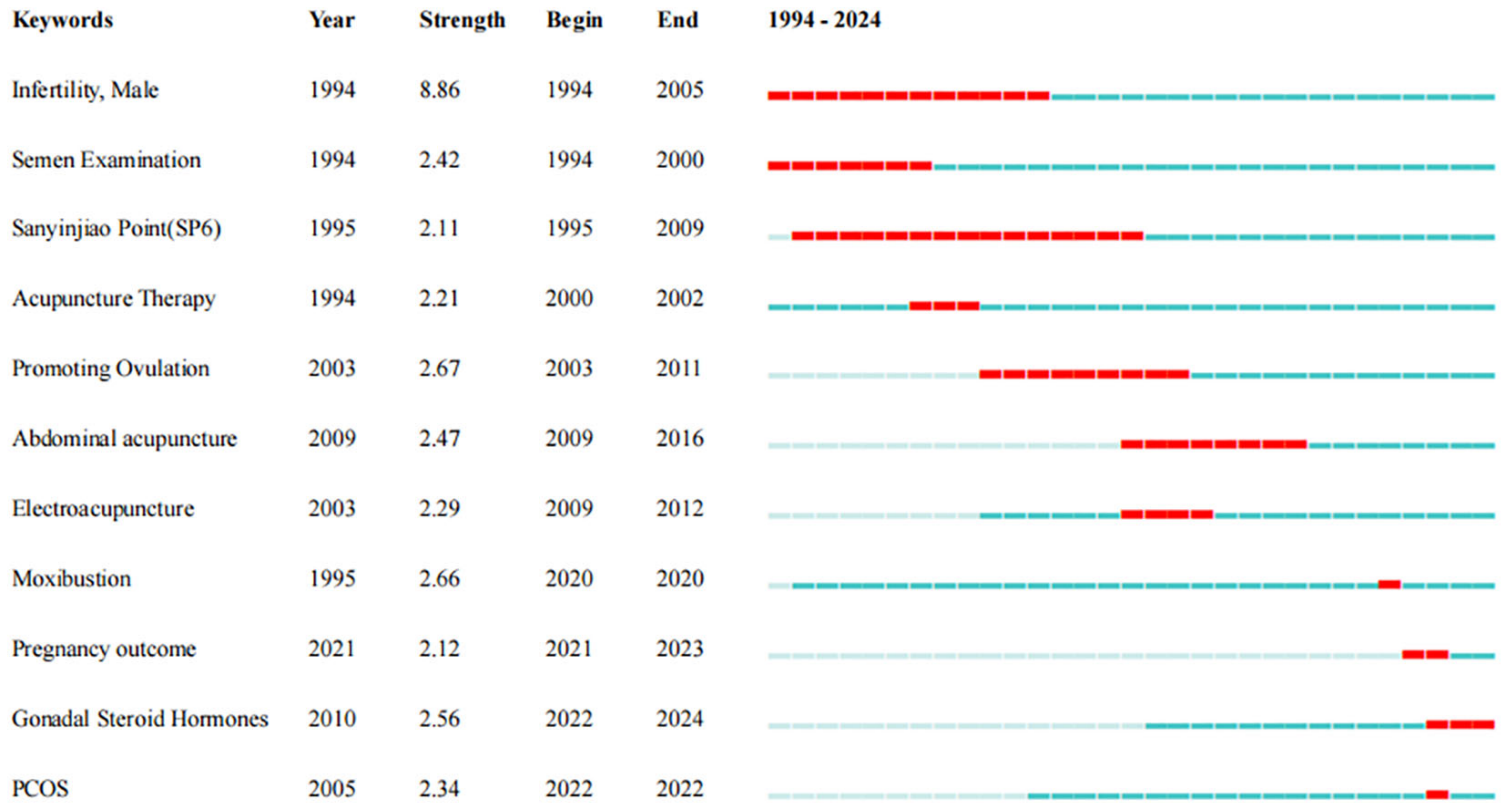
Citation burst on keyword.

Citation burst on keywords can reflect the research hotspots in this field at different times. This study has obtained 11 burst terms (see **Figure 5**). The burst term “male infertility” lasted up to 11 years, indicating that acupuncture for male infertility was a hotspot in the early research stage, while the burst term “Sanyinjiao” reflected that this acupoint was frequently used for infertility. Abdominal acupuncture, electroacupuncture, and moxibustion reflected the researchers’ exploration of various acupuncture therapies at different times. In the recent 2 years from 2021 to 2023, the acupuncture for infertility caused by polycystic ovary syndrome (PCOS) was a hotspot research direction, mainly focusing on the impact of acupuncture on pregnancy outcomes and sex hormones.

Among the 274 primary studies, there were 40 (14.60%) cases of male infertility and 234 (85.40%) cases of female infertility. In the studies of male infertility, six kinds of diseases or causes were reported, including 24 (60.00%) idiopathic male infertility, 4 (10.00%) immune infertility, and 5 (12.50%) infertility caused by other diseases or causes, such as sexual dysfunction, varicocele, and genitourinary tract infection. Six studies (17.50%) did not report the specific disease or cause of male infertility and one (2.5%) study did not report in detail. As for female infertility, 20 kinds of diseases or causes were reported, with PCOS being the most common disease involving 80 (34.19%) studies. There were 19 (8.12%) studies about luteinized unruptured follicle syndrome (LUFS), 46 (19.66%) studies about ovulation disorder, 13 (5.56%) studies about block of fallopian tube, 8 studies about thin endometrium, 5 studies (2.14%) about luteal phase defect, and 28 (11.96%) studies about other diseases or causes such as heterotopia endometriosis, adenomyosis, hyperprolactinemia, decreased ovarian reserve function, ovarian insufficiency, premature ovarian failure, posterior uterus, serum anti-sperm antibody positive infertility, follicular dysplasia, immunological infertility, endocrine infertility, and unexplained infertility. Thirty studies (12.82%) did not report the specific disease or cause of female infertility and five (2.14%) studies did not report in detail.

**Figure 6:**
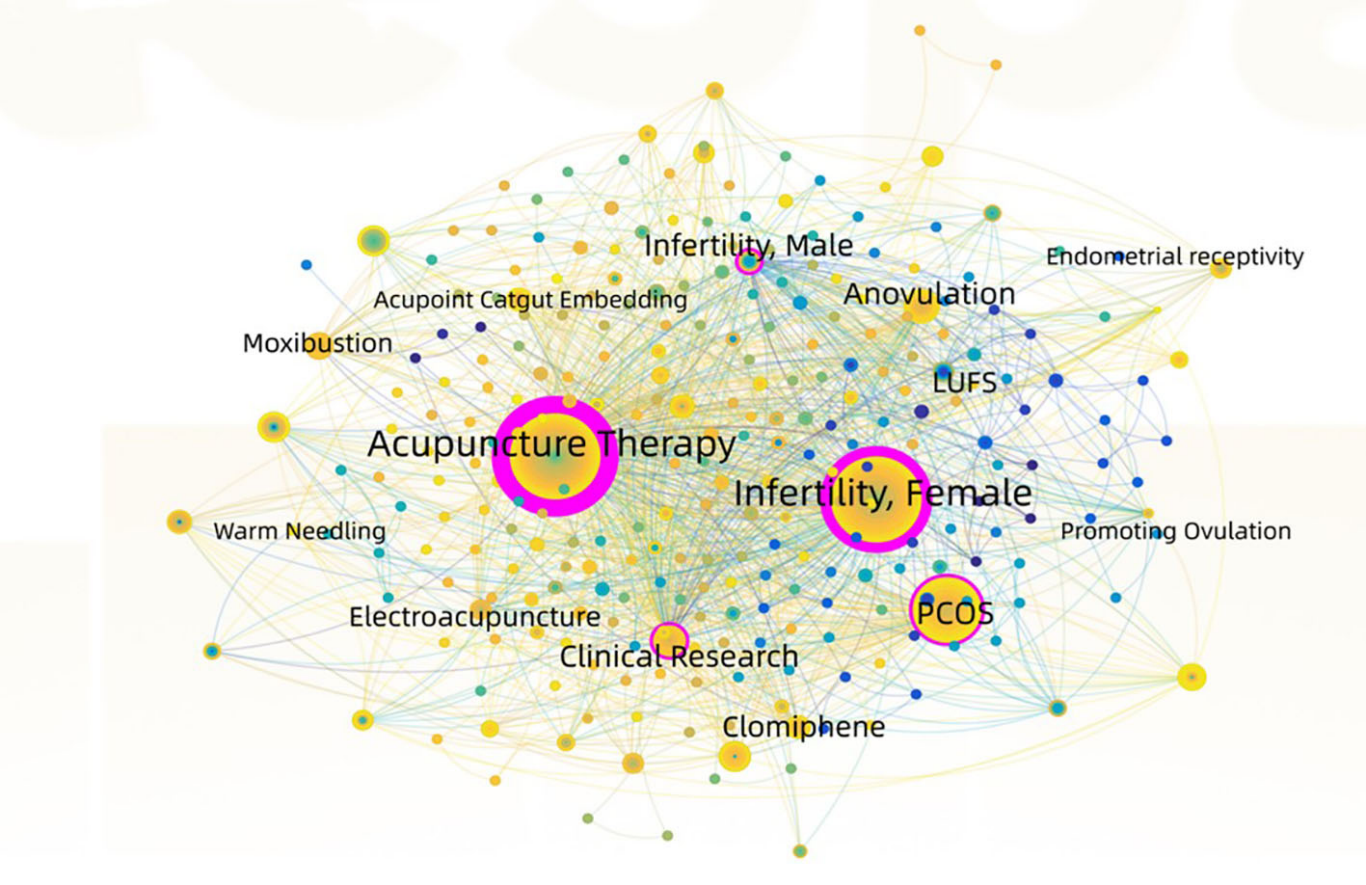
Co-occurrence map of keyword.

The results of keyword co-occurrence analysis showed that the current research highlights on acupuncture for infertility focused on female infertility caused by PCOS, ovulation disorder, and LUFS, and the acupuncture interventions with high frequency included acupuncture alone, moxibustion, acupoint catgut embedding, electroacupuncture, and warm acupuncture (see **Table 2**, **Figure 6**).

**Table 2:** Top 14 keywords.

Number	Keywords	Count	Centrality
1	Infertility, female	141	0.56
2	Acupuncture therapy	141	0.83
3	Polycystic ovary syndrome	71	0.16
4	Clinical research	43	0.16
5	Anovulation	40	0.06
6	Infertility, male	25	0.15
7	Moxibustion	20	0.07
8	Clomiphene	18	0.02
9	Acupoint catgut embedding	17	0.08
10	Electroacupuncture	16	0.03
11	Luteinized unruptured follicle syndrome	14	0.03
12	Promoting ovulation	11	0.03
13	Endometrial receptivity	10	0.03
14	Warm needling	10	0

### 3.3 Interventions

#### 3.3.1 Types of acupuncture vs. comparison

The types of acupuncture vs. comparison are listed in detail in **Table 3**. In 192 RCTs, the types of comparison were classified into 13 categories, among which the type of acupuncture combined with Western medicine vs. Western medicine was the most common type, with 96 studies (50%) in total. This was followed by 76 studies (39.58%) comparing acupuncture alone with Western medicine.

**Table 3:** Comparison types of acupuncture versus control group.

Study types	Comparison types	Number of studies
RCT	Acupuncture combined with Western medicine vs. Western medicine	96
	Acupuncture vs. Western medicine	76
	Acupuncture combined with surgery vs. Surgery	5
	Acupuncture combined with Western medicine vs. Western medicine vs. Acupuncture	4
	Acupuncture vs. Sham acupuncture	3
	Acupuncture vs. Western medicine vs. Condom isolation	1
	Acupuncture combined with Western medicine vs. Fake acupuncture combined with Western medicine vs. Acupuncture	1
	Acupuncture combined with behavioral therapy combined with Western medicine vs. Western medicine	1
	Acupuncture combined with infrared irradiation vs. Blank	1
	Acupuncture combined with Western medicine and endovascular therapy vs. Western medicine	1
	Acupuncture combined with guidance vs. General treatment	1
	Acupuncture combined with lifestyle intervention and Western medicine vs. lifestyle intervention and Western medicine	1
	Acupuncture combined with core muscle rehabilitation exercise and Western medicine vs. Western medicine	1
CCT	Acupuncture combined with Western medicine vs. Western medicine	2
	Acupuncture vs. Western medicine	1
	Acupuncture combined with hysterosalpingography vs. Hysterosalpingography	1
	Western medicine A combined with acupuncture vs. Western medicine A combined with B vs. Western medicine A combined with Western medicine C	1
Matched control study	Acupuncture combined with Western medicine vs. Western medicine	1
Cohort study	Acupuncture vs. Western medicine	1
	Acupuncture combined with Western medicine vs. Western medicine	2

Western medicine: clomiphene citrate, letrozole, human chorionic gonadotropin, urinary gonadotropin, human menopausal gonadotropin, diethylstilbestrol, estradiol valerate, deoxyprogesterone, progesterone, progesterone, progesterone acetate, progesterone, estradiol/estradiol flexor progesterone, methylprednisolone, prednisone, metformin, bromocriptine mesylate, and aspirin. Operation: pelvic adhesiolysis, tubal fluid opening, tubal umbrella endoplasty, tubal umbrella endostomy, and ultrasonic crystal oxygen salpingography.

#### 3.3.2 Acupoint prescriptions

Among 274 studies, the prescription of acupoint could be classified into three categories: (1) 202 (73.72%) studies with a fixed acupoint prescription (same acupoints for each patient); (2) 66 (24.09%) studies with a semi-fixed acupoint prescription (fixed acupoints combined with syndrome differentiation) according to the symptoms and characteristics of the disease; and (3) 6 (2.19%) studies with individualized selection acupoints, according to the characteristics of the disease.

There were four kinds of sources of acupoint prescription: (1) 9 studies (3.28%) were based on the relevant acupuncture textbooks; (2) 1 study (0.36%) was based on the relevant clinical guideline; (3) 14 studies (5.11%) were based on the experience of veteran TCM experts; and (4) 4 studies (1.46%) were based on personal clinical experience. A total of 246 studies (89.78%) did not explicitly report the source of the acupoint prescription.

#### 3.3.3 Frequency and course of acupuncture

Among the 274 studies, 202 (73.72%) studies provided acupuncture three times or more per week, 22 (8.03%) studies provided acupuncture twice a week, 25 (9.12%) studies provided acupuncture once a week, and 27 (9.85%) studies did not clearly report the frequency of acupuncture. Of the 234 studies on female infertility, the course of acupuncture was 3 months or three menstrual cycles in 128 (54.70%) studies, 6 months or six menstrual cycles in 16 (6.84%) studies, and 56 (23.93%) studies did not explicitly report the course of acupuncture. Of the 40 studies on male infertility, 10 (25%) studies had a 3-month acupuncture course and 23 (57.5%) did not explicitly report this information.

### 3.4 Qualification of acupuncturist

There was just one RCT published in English that reported training the acupuncturist at the beginning of the study.

### 3.5 Outcomes and results

Of the 40 studies on male infertility, 12 studies (30%) defined pregnancy rates as outcome; 14 studies (35%) used sperm motility, quantity, and fragmentation as outcomes; and 14 studies (35%) included these two outcomes.

Of the 234 female studies, only 1 used both pregnancy rate and live birth rate as outcome; the remaining 233 studies only reported pregnancy rate as outcome.

As for the results, among 192 RCTs, 147 RCTs (76.56%) had positive results (*p* < 0.05) and concluded that the acupuncture group may have a better effect than the control group, and 28 RCTs (14.58%) reported negative results (*p* > 0.05) and concluded that there were no differences between acupuncture groups and control groups. There were 17 studies that did not report *p*-values. Of the five included CCTs, four studies reported positive results while the rest reported negative results. The three cohort studies reported positive results; one matched control study reported negative results.

### 3.6 Funding sources

Seventy-three studies reported funding sources, including 12 (16.44%) national projects, 54 (73.97%) provincial projects, and 7 (9.60%) college and university projects.

### 3.7 Adverse events

There were 34 studies providing adverse event reports, among which 8 studies reported no adverse events after acupuncture treatment and 26 studies reported adverse events including mild bleeding, sluggishness, dizziness, fatigue, nausea, vomiting, abdominal discomfort, mild ovarian stimulation, threatened abortion, weight gain, and visual disturbances.

### 3.8 Follow-up

Of the 274 studies, only 70 (25.55%) reported follow-up information after completion of acupuncture, with a minimum follow-up period of 3 weeks and a maximum follow-up period of 3 years. The remaining 204 (74.45%) studies did not mention follow-up after the trials.

### 3.9 Meta-analysis of acupuncture for LUFS infertility

Among the seven included meta-analyses, acupuncture for infertility caused by PCOS, ovulation dysfunction, and anovulatory have been published in the recent 4 years (21–24); the details about the seven included meta-analyses are provided in **Supplementary Table S5**. Except for LUFS, the number of RCTs for male infertility and female infertility caused by other diseases were less than three; therefore, we only conducted meta-analysis of acupuncture for LUFS infertility.

#### 3.9.1 Characteristics of the included 17 RCTs

There were 17 included RCTs on female infertility caused by LUFS (25–41). All studies were published in Chinese. There were 1,099 patients involved, with 553 in the intervention group and 546 in the control group. The minimum sample size in the study was 50, and the maximum was 84. One study (37) did not report diagnostic criteria. Only three studies (29, 30, 32) reported follow-up time, while the remaining studies did not report follow-up information. The basic characteristics of the included RCTs are shown in **Table 4**.

**Table 4:** Characteristics of included RCTs of acupuncture for luteinized unruptured follicle syndrome.

Included studies	Sample size	Age	Treatment group interventions	Control group interventions	Course of treatment	Outcome
(41)	T: 36 C: 36	T:/ C:/	Acupuncture + HCG	HCG	4 weeks	Pregnancy rate
(40)	T: 30 C: 30	T: 32.51 ± 2.75 C: 31.79 ± 3.62	Acupuncture	HCG	3 menstrual cycles	Pregnancy rate
(38)	T: 36 C: 36	T: 23–38 C: 22–38	Acupuncture + HCG	HCG	3 menstrual cycles	Pregnancy rate
(39)	T: 43 C: 41	T:/ C:/	Acupuncture + HCG + letrozole	HCG + letrozole	3 menstrual cycles	Pregnancy rate
(35)	T: 30 C: 30	T: 29.30 ± 3.67 C: 28.37 ± 3.79	Acupuncture	HCG	No report	Pregnancy rate
(37)	T: 33 C: 33	T: 23–40 C: 23–40	Acupuncture	HCG	3 menstrual cycles	Pregnancy rate
(36)	T: 40 C: 40	T: 25.8 ± 2.56 C: 24.8 ± 2.16	Acupuncture	HCG	3 menstrual cycles	Pregnancy rate
(34)	T: 30 C: 30	T: 28.27 ± 3.84 C: 28.85 ± 2.13	Acupuncture	HCG	3 menstrual cycles	Pregnancy rate
(33)	T: 32 C: 32	T: 29.59 ± 3.44 C: 31.22 ± 12.79	Acupuncture	HCG	3 menstrual cycles	Pregnancy rate
(32)	T: 30 C: 30	T: 31.87 ± 3.58 C: 31.77 ± 3.63	Acupuncture + HCG	HCG	1 month	Pregnancy rate
(31)	T: 30 C: 30	T:/ C:/	Acupuncture	HCG	No report	Pregnancy rate
(29)	T: 33 C: 32	T: 29.39 ± 4.39 C: 29.22 ± 4.15	Acupuncture	HCG	3 months	Pregnancy rate
(30)	T: 30 C: 26	T: 30.27 ± 3.44 C: 30.07 ± 2.89	Acupuncture	HCG	3 menstrual cycles	Pregnancy rate
(28)	T: 25 C: 25	T: 30.48 ± 4.03 C: 29.71 ± 3.05	Acupuncture	HCG	3 menstrual cycles	Pregnancy rate
(27)	T: 25 C: 25	T: 30.88 ± 3.37 C: 31.60 ± 4.56	Acupuncture	No treatment	No report	Pregnancy rate
(26)	T: 40 C: 40	T: 29.18 ± 2.63 C: 29.00 ± 2.71	Acupuncture + HCG + endovascular therapy	HCG	3 menstrual cycles	Pregnancy rate
(25)	T: 30 C: 30	T:/ C:/	Acupuncture + HCG	HCG	3 menstrual cycles	Pregnancy rate

T, treatment group; C, the control group; HCG, human chorionic gonadotropin.

#### 3.9.2 Quality assessment of the included RCTs

**Figure 7:**
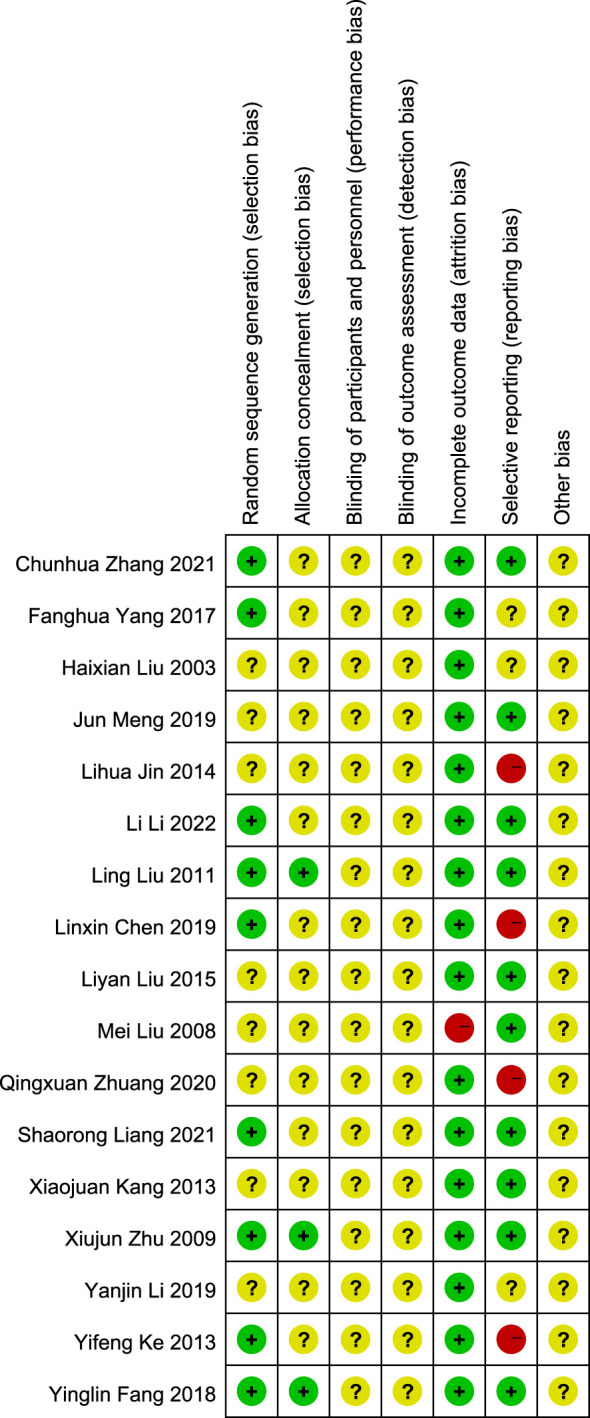
Risk of bias 17 included RCTs.

**Figure 8:**
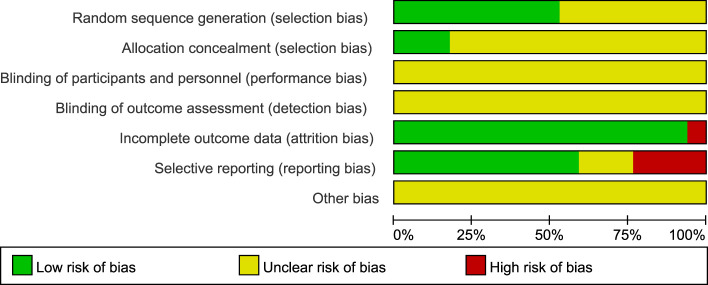
Risk of bias summary of 17 included RCTs.

The risk of bias on primary outcome in 17 RCTs is summarized in **Figures 7** and **8**. Nine RCTs used the random number table and computer software to generate random sequences, which was considered to have a low risk of bias. Eight RCTs (25, 26, 29, 31, 32, 36, 37, 39) only mentioned “random” but did not report the details, and the risk of bias was considered to be unclear. Three RCTs reported the use of opaque envelopes as their random sequence concealment methods (27, 28, 34), which were considered to have a low risk of bias. Others did not report this information and were considered to have unclear risk of bias. All studies did not report the use of blinding, and the risk of bias was considered to be unclear. As for incomplete outcome data, one RCT (26) reported that the number of dropouts and deletions exceeded 20% of the total sample size, indicating a high risk of bias. Other studies were evaluated as having a low risk of bias. Four RCTs (30, 31, 35, 39) did not report predetermined outcomes and were evaluated as having a high risk of bias; three RCTs (25, 33, 36) did not report the study protocol and registration information and were considered to have unclear risk of bias; the remaining 10 RCTs were considered to have a low risk of bias. Because of insufficient reporting on items such as random allocation methods, random sequence concealment, and implementation of blinding in the included studies, as well as a high risk of methodological bias, the overall methodological quality of the included RCTs was not high.

#### 3.9.3 Pregnancy rate of acupuncture for LUFS infertility

##### 3.9.3.1 Acupuncture vs. human chorionic gonadotropin

**Figure 9:**
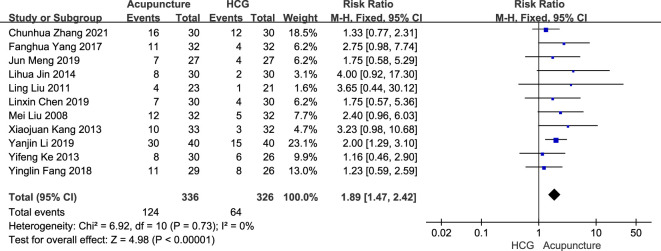
Forest plots of the pregnancy rate comparison between acupuncture and HCG.

The fixed-effects model meta-analysis showed that compared with HCG therapy, acupuncture therapy a resulted in a higher pregnancy rate [RR = 1.89, 95% CI (1.47, 2.42), 11 RCTs, 662 participants, *p* < 0.00001] (see **Figure 9**).

##### 3.9.3.2 Acupuncture + HCG vs. HCG

**Figure 10:**
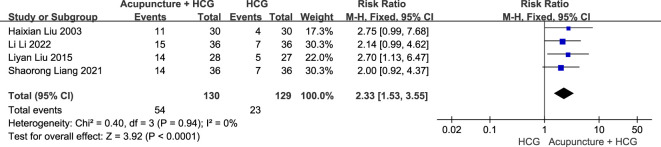
Forest plots of the pregnancy rate comparison between acupuncture combined with HCG and HCG alone.

The fixed-effects model meta-analysis showed that compared with HCG therapy alone, acupuncture combined with HCG therapy resulted in a higher pregnancy rate [RR = 2.33, 95% CI (1.53, 3.55), four RCTs, 259 participants, *p* < 0.0001] (see **Figure 10**).

##### 3.9.3.3 Acupuncture vs. no treatment

One RCT (27) reported that compared with no treatment, acupuncture resulted in a higher pregnancy rate [RR = 22.12, 95% CI (1.39, 353.09), one RCT, 47 participants, *p* = 0.03].

##### 3.9.3.4 Acupuncture + HCG + letrozole vs. HCG + letrozole

For the comparison between acupuncture combined with HCG plus letrozole and HCG plus letrozole, the result showed no significant difference on the pregnancy rate (39) [RR = 1.56, 95% CI (0.84, 2.89), one RCT, 84 participants, *p* = 0.16].

#### 3.9.4 Publication bias

**Figure 11:**
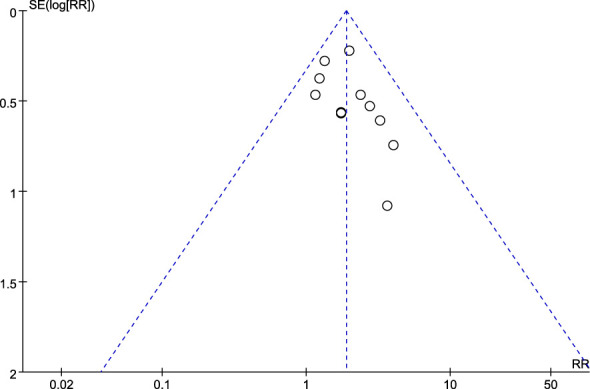
Funnel plot.

Funnel plots were drawn to explore the possibility of publication bias for the 17 RCTs. The scattered point distribution in the funnel plots was basically symmetrical (see **Figure 11**).

## 4 Discussion

### 4.1 Main findings

Acupuncture can be used for infertility caused by various male and female factors. Current research highlights on acupuncture for infertility focused on female infertility caused by PCOS, ovulation disorder, and LUFS, while studies on male infertility and female infertility caused by blockage in the fallopian tube, thin endometrium, and luteal phase defect, among others, were insufficient. Current existing evidence of acupuncture on infertility indicated that the RCTs were still the main study type, but the information on the funding source for these RCTs was not evident. There was a lack of studies with large sample sizes and the acupoints tended to follow a fixed protocol (73.5%) (but the source of the acupoint protocol was not clear). The frequency and course of acupuncture varied from once to thrice a week and from 3 months to more than 6 months, respectively. Adverse events reported included mild ovarian irritation and potential early signs of miscarriage (some uncomfortable local pain). The contribution rate of different authors to this discipline varies depending on their publication volume, the core authors with higher contribution rates gather into a core author group, and no core author group has yet been formed in this field. Because the centrality of different institutions has not reached the target value 0.1, a cooperative network between different institutions has not been formed.

As for the acupuncture intervention itself, it is a complex intervention; the acupoints, frequency, and course of intervention differ for different patients according to different conditions. In particular, when it comes to pregnancy safety, professional qualified acupuncturists are required. No study focused on health economic analyses, and most RCTs with positive results did not explicitly propose their hypothesis (superiority) but concluded that the acupuncture intervention group was more clinically effective than the control group. For the large sample size confirmatory clinical studies, examination of statistical differences between two groups is critical to substantiate the results and it is important to establish an appropriate hypothesis and aim at the design stage of the study. Superiority, non-inferiority, or equivalence clinical trials should be clearly described for each study in order to correctly interpret the results (42).

### 4.2 Limitations of this study

This study was conducted according to the guidelines of scoping reviews. The quality of included studies was not assessed as this is not required for scoping reviews. A previous scoping review on acupuncture for IVF has been published; thus, we excluded studies that combined acupuncture with ART, and the outcomes were limited to pregnancy rate, live birth rate, or semen parameters. We only searched seven databases, namely, three English databases and four Chinese databases, and we will consider including additional relevant databases such as Web of Science to enhance the comprehensiveness of this study when we update this scoping review in the coming years. This summary of current evidence does not reflect the effectiveness of acupuncture on other parameters such as menstruation, ovulation disorder, and sperm motility. Other exclusions involved studies that combined acupuncture and herbal medicine or used herbal medicine as the control group, while herbal medicine is also an alone or adjuvant treatment for infertility in clinical practice.

### 4.3 Implications for future research

Acupuncture is widely used all over the world, and the increase in funding for acupuncture clinical studies has provided opportunities for current researchers to produce more high-quality primary clinical evidence (43). As we have summarized in this manuscript, there are currently at least 25 kinds of conditions or causes about male and female infertility clinical studies has been published, are they all the dominant populations of acupuncture? Pilot studies are still required to screen acupoint regimens, frequency, and course of acupuncture intervention and even dominant populations based on syndrome pattern differentiation and its treatment of acupuncture. As for real-world studies with a large sample size, researchers should consider the heterogeneity between participants. Future studies on acupuncture should not ignore the complexity of acupuncture, its different forms of delivery, techniques, dependence on expertise, use of combined therapies, and highly personalized treatment regimens (44). Researchers should also consider the inclusion of a qualitative interview in their study design. Meanwhile, health economics analysis of acupuncture on infertility could identify whether there are cost savings if the intervention was more widely available. Different countries have different medical systems and policies. Appropriate research design should be considered such as superiority, non-inferiority, or equivalence clinical trials. Moreover, researchers should also follow the reporting guidelines according to different study types.

## 5 Conclusions

This study identified and summarized the current clinical evidence of acupuncture for infertility. Acupuncture may potentially be used for treating various male and female infertility factors. Current research highlights on acupuncture for infertility focused on female infertility caused by PCOS, ovulation disorder, and LUFS, while studies on male infertility and female infertility caused by blockage in the fallopian tube, thin endometrium, and other factors were insufficient. Despite the large number of RCTs in this field, larger confirmatory clinical studies with appropriate hypothesis evidence on acupuncture for infertility resulting in natural conception are still lacking as the research hypotheses for most studies were unclear and most studies were exploratory in nature with small sample sizes.

## Data availability statement

The original contributions presented in the study are included in the article/**Supplementary Material**. Further inquiries can be directed to the corresponding authors.

## Author contributions

ZT: Conceptualization, Methodology, Project administration, Writing – original draft. CZ: Data curation, Software, Visualization, Writing – original draft. XL: Writing – review & editing, Methodology, Supervision. SY: Formal analysis, Writing – review & editing. YH: Data curation, Writing – review & editing. AS: Data curation, Writing – review & editing. FY: Data curation, Writing – review & editing. TP: Data curation, Writing – review & editing. JZ: Data curation, Writing – review & editing. YM: Writing – review & editing, Data curation. NR: Conceptualization, Writing – review & editing. PB: Conceptualization, Writing – review & editing, Project administration. WG: Conceptualization, Methodology, Supervision, Writing – review & editing.
