# Corrigendum: Trends in acupuncture for infertility: a scoping review with bibliometric and visual analysis

**DOI:** 10.3389/fendo.2024.1444444

**Published:** 2024-06-28

**Authors:** Ziyu Tian, Chongyang Zhang, Xing Liao, Sihong Yang, Yuying Hong, Anni Shi, Fei Yan, Ting Pan, Jiajia Zhang, Yan Meng, Nicola Robinson, Peng Bai, Weijuan Gang

**Affiliations:** ^1^ Dongzhimen Hospital, Beijing University of Chinese Medicine, Beijing, China; ^2^ Institute of Acupuncture and Moxibustion, China Academy of Chinese Medical Sciences, Beijing, China; ^3^ Third Affiliated Hospital, Beijing University of Chinese Medicine, Beijing, China; ^4^ Center for Evidence Based Chinese Medicine, Institute of Basic Research in Clinical Medicine, China Academy of Chinese Medical Sciences, Beijing, China; ^5^ China Center for Evidence Based Traditional Chinese Medicine, China Academy of Chinese Medical Sciences, Beijing, China; ^6^ School of Acupuncture-Moxibustion and Tuina, Beijing University of Chinese Medicine, Beijing, China; ^7^ Department of Gynecology, Dongzhimen Hospital, Beijing University of Chinese Medicine, Beijing, China; ^8^ Department of Acupuncture and Moxibustion, Beijing Longfu Hospital, Beijing, China; ^9^ Centre for Evidence-Based Chinese Medicine, Beijing University of Chinese Medicine, Beijing, China; ^10^ Institute of Health and Social Care, London South Bank University, London, United Kingdom

**Keywords:** infertility, natural conception, acupuncture, scoping review, bibliometric and visual analysis

In the published article, there was an error regarding the affiliation for Ziyu Tian. As well as having affiliation 2, Institute of Acupuncture and Moxibustion, China Academy of Chinese Medical Sciences, they should also have *1, Dongzhimen Hospital, Beijing University of Chinese Medicine, Beijing, China*.

The authors apologize for this error and state that this does not change the scientific conclusions of the article in any way. The original article has been updated.

