# Supplementary material for: Trends in acupuncture for infertility: a scoping review with bibliometric and visual analysis
Source: Front Endocrinol (Lausanne). 2024 Jun 4;15:1351281. doi: 10.3389/fendo.2024.1351281 (PMC11183275; doi:10.3389/fendo.2024.1351281)
Supplement: Supplementary file 1 [file Table_1.docx]

Supplementary Material

# Supplementary Tables

**Table S1： PubMed searching strategies**

| #1 | Acupuncture[MeSH Terms] OR Pharmacopuncture[Title/Abstract] OR "Acupuncture Therapy"[MeSH Terms] OR "Acupuncture Treatment"[Title/Abstract] OR "Pharmacoacupuncture Treatment"[Title/Abstract] OR "Pharmacoacupuncture Therapy"[Title/Abstract] OR Acupotomy[Title/Abstract] OR Moxibustion[MeSH Terms] OR "Auricular Acupuncture"[Title/Abstract] OR "Ear Acupuncture"[Title/Abstract] OR "Acupuncture Points"[MeSH Terms] OR Acupoint[Title/Abstract] OR "Cupping Therapy"[MeSH Terms] OR "Cupping Treatment"[Title/Abstract] OR "Warm needling"[Title/Abstract] OR "fire needling"[Title/Abstract] OR "plum-blossom needle therapy"[Title/Abstract] OR "scalp acupuncture"[Title/Abstract] OR "Three-edged needle"[Title/Abstract] OR "Wrist ankle needle"[Title/Abstract] OR "abdominal acupuncture"[Title/Abstract] OR "intradermal needle"[Title/Abstract] OR "dermal needle"[Title/Abstract] OR "scraping therapy"[Title/Abstract] OR "acupoint application"[Title/Abstract] OR "acupoint catgut embedding"[Title/Abstract] |
| --- | --- |
| #2 | Infertility[MeSH Terms] OR "Reproductive Sterility"[Title/Abstract] OR Subfertility[Title/Abstract] OR (Infertility, Female[MeSH Terms] OR "Female Infertility"[Title/Abstract] OR "Postpartum Sterility"[Title/Abstract] OR "Female Subfertility"[Title/Abstract] OR "Female Sterility"[Title/Abstract] OR Pregnancy[MeSH Terms] OR Gestation[Title/Abstract] OR "Pregnancy Rate"[MeSH Terms] OR "Live Birth Pregnancy Rate"[Title/Abstract] OR "Live Birth"[MeSH Terms] OR "Pregnancy Outcome"[MeSH Terms] OR Infertility, Male[MeSH Terms] OR "Male Infertility"[Title/Abstract] OR "Male Sterility"[Title/Abstract] OR "Male Subfertility"[Title/Abstract] OR Aspermia[MeSH Terms] OR Asthenozoospermia[MeSH Terms] OR "Astheno Teratozoospermia"[Title/Abstract] OR "Asthenoteratozoospermia"[Title/Abstract] OR Azoospermia[MeSH Terms] OR Oligospermia[MeSH Terms] OR Hypospermatogenesis[Title/Abstract] OR "Low Sperm Count"[Title/Abstract] OR Oligoasthenoteratozoospermia[Title/Abstract] OR Oligozoospermia[Title/Abstract] OR "Sertoli Cell-Only Syndrome"[MeSH Terms] OR "Del Castillo Syndrome"[Title/Abstract] OR "Germinal Cell Aplasia"[Title/Abstract] OR Teratozoospermia[MeSH Terms] OR "Abnormal Spermatozoa"[Title/Abstract] OR Teratospermia[Title/Abstract] OR Globozoospermia[Title/Abstract] |
| #3 | #1 AND #2 |
| #4 | "Fertilization in Vitro"[MeSH Terms] OR "Test Tube Fertilization"[Title/Abstract] OR "Test-Tube Baby"[Title/Abstract] OR "Embryo Transfer"[MeSH Terms] OR "Blastocyst Transfer"[Title/Abstract] OR "Tubal Embryo Transfer"[Title/Abstract] OR "Tubal Embryo Stage Transfer"[Title/Abstract] |
| #5 | #3 NOT #4 |

**Table S2： Characteristics of 192 RCTs**

| ID | Author(same study ID, use -1; -2) | Year of publication | Journal/Conference Name/Dissertation Awarding Unit | Type of publication ^a^ | Language^b^ | Fund support | Type of Study | Research purposes | Sample size | Diseases or causes of female infertility | Diseases or causes of male infertility |
| --- | --- | --- | --- | --- | --- | --- | --- | --- | --- | --- | --- |
| 1 | Wang Fan | 2024 | Journal of practical Chinese medicine | 1 | 1 | No | RCT | To observe the effect of Tiaoretongdu acupuncture combined with Tongyuan acupuncture in treating infertility of polycystic ovary syndrome (PCOS). | 82 | Polycystic ovary syndrome |  |
| 2 | Wang Xiumei; Sun Sa; Zhang Xiuhong; Yuan Hongli; Liu Huan; Zhou Yu | 2024 | Shaanxi traditional Chinese medicine | 1 | 1 | Provincial and ministerial projects | RCT | To explore the clinical effect of acupuncture and moxibustion on thin endometrial infertility patients based on the theory of "left host cell palace". | 80 | Thin endometrium |  |
| 3 | Chen Jiao; Fan Huaying; Zeng Jiuzhi; Liu Yu; Hu Jinqun; Zhou Minqing; Huang Juan; Liang Fanrong | 2024 | Chinese Journal of Traditional Chinese Medicine | 1 | 1 | Provincial and ministerial projects | RCT | To investigate the clinical effect of electroacupuncture cycle therapy combined with Clomiphene citrate in the treatment of polycystic ovary syndrome (PCOS). | 116 | Polycystic ovary syndrome |  |
| 4 | He Qida; Fang Maolin; Zhong Zhenghong; Tong Ziyan; Chen Min | 2023 | Chinese Journal of Traditional Chinese Medicine | 1 | 1 | Provincial and ministerial projects | RCT | To study the clinical effect of electroacupuncture combined with moxibustion on infertility of ovarian resistance syndrome (ROS). | 60 | Ovarian resistance syndrome |  |
| 5 | Yi Chendi; Sun Yun; Li Ting | 2023 | Chinese medicine science and technology | 1 | 1 | Provincial and ministerial projects | RCT | To investigate the clinical effect of electroacupuncture combined with pelvic floor muscle electrical stimulation on thin endometrial infertility and its influence on endometrial blood flow. | 90 | Thin endometrium |  |
| 6 | Fan E; Yang Lina; Cui Weixia; Wei Qiaoling; Liu Juanjuan; Luo Caifeng; Yang Yange | 2023 | Clinical research and experience | 1 | 1 | Provincial and ministerial projects | RCT | To observe the clinical effect of Dunhuang heavy moxibustion combined with warm tong acupuncture on infertility of spleen-kidney-yang deficiency type polycystic ovary syndrome. | 36 | Polycystic ovary syndrome |  |
| 7 | Wang Ke; Zhang Wanlong; Sun Yini; Zhu Ling; Li Yi | 2023 | Shanghai Journal of Acupuncture | 1 | 1 | Provincial and ministerial projects | RCT | To observe the effects of abdominal acupuncture combined with Clomiphene citrate tablet on endometrial receptivities, anxiety state and pregnancy outcome of ovulation disorder infertility patients with liver depression and kidney deficiency. | 70 | Ovulation disturbance |  |
| 8 | Ren Lijuan; Li Hongying; Xie Hongying; Zhang Zhenzhen; Xiao Xiuzhen | 2023 | Chinese modern doctor | 1 | 1 | Provincial and ministerial projects | RCT | To investigate the clinical value of transvaginal ultrasonography in the treatment of infertility of polycystic ovarian syndrome (PCOS) by combination of traditional Chinese and Western medicine (acupuncture combined with Western medicine). | 100 | Polycystic ovary syndrome |  |
| 9 | Wang Shujuan; Yang Peihong; Jia Pingping | 2023 | Journal of Practical Chinese Medicine Internal Medicine | 1 | 1 | Provincial and ministerial projects | RCT | To explore the effect of life style intervention combined with acupuncture point embedding on infertility patients with polycystic ovary syndrome. | 80 | Polycystic ovary syndrome |  |
| 10 | Mao Runti; Li Xiulan; Mo Yujun; Chen Junying; Luo Shuxiong | 2023 | Modern medicine and health research | 1 | 1 | No | RCT | To analyze the effects of "soothing the liver and strengthening the spleen, tonifying the kidney and supplementing the essence" acupuncture on endometrial receptivity, follicle development and serum luteinizing hormone (LH), estradiol (E2) and follicle-stimulating hormone (FSH) levels in patients with ovulation dysfunction infertility. | 80 | Ovulation disturbance |  |
| 11 | Yang Ting; Tan Jiaojiao; Yang Jing | 2023 | Hebei traditional Chinese medicine | 1 | 1 | No | RCT | To observe the effects of warm acupuncture and moxibustion combined with conventional western medicine on ovarian function, pregnancy and sex hormone in infertility patients with polycystic ovary syndrome. | 121 | Polycystic ovary syndrome |  |
| 12 | Gu Ying; Dai Linjun | 2023 | Zhejiang Journal of Traditional Chinese Medicine | 1 | 1 | Provincial and ministerial projects | RCT | Not report | 60 | Thin endometrium |  |
| 13 | Wang Yunmeng; Wang Ruixia; Wang Qianqian | 2023 | Chinese folk therapy | 1 | 1 | Provincial and ministerial projects | RCT | To observe the effect of acupoint embedding on endometrium and pregnancy outcome of infertile patients with kidney deficiency and blood stasis polycystic ovary syndrome (PCOS). | 80 | Polycystic ovary syndrome |  |
| 14 | Wang Ke; Zhang Wanlong; Li Yi | 2023 | Shanghai Journal of Acupuncture | 1 | 1 | Bureau level project | RCT | To observe the effect of abdominal acupuncture combined with oral Clomiphene citrate and estradiol valerate tablets on the pregnancy outcome of ovulation dysfunction infertility patients. | 60 | Ovulation disturbance |  |
| 15 | Yuan Shixin; Zhou Yanli; Liu Jiangtao; Zhou Hongtu; Li Wenzhong; Qiao Mingmin | 2022 | Journal of Traditional Chinese Medicine | 1 | 1 | Provincial-level project | RCT | To observe the clinical effect of acupuncture on kidney deficiency and blood stasis type premature ovarian failure. | 60 | Premature ovarian failure |  |
| 16 | Guan Yujie | 2022 | Asia-pacific traditional medicine | 1 | 1 | None | RCT | To compare the effect of acupuncture therapy and ovulation stimulating needle in the treatment of follicular dysplasia. | 60 | Follicular dysplasia |  |
| 17 | Tang Xinli | 2022 | Health friend | 1 | 1 | None | RCT | Failure to report | 100 | Not reported |  |
| 18 | Wang Jingxiao; Li Jia; Shen Weidong | 2022 | Chinese Journal of Family Planning | 1 | 1 | None | RCT | To investigate the effect of acupuncture combined with clomiphene in the treatment of polycystic ovary syndrome (PCOS) | 120 | Polycystic ovary syndrome |  |
| 19 | Wang Wei; Huang Yong | 2022 | Chinese Journal of Family Planning | 1 | 1 | None | RCT | To explore the therapeutic effect of Baliao acupuncture combined with dehydroepiandrosterone (DHEA) on infertility patients with early-onset ovarian insufficiency (POI) | 108 | Ovarian insufficiency |  |
| 20 | Sun Junjian; Xie Henghui | 2022 | Clinical journal of Chinese medicine | 1 | 1 | District subject | RCT | To observe the clinical effect of acupuncture on ovulation sterility of small follicle in kidney Qi deficiency syndrome. | 100 | Small follicles ovulate |  |
| 21 | Wang Yu; Highly esteemed letter; He Hui; Ma Hongli; Cluster crystal; Yang Xinming; Wu Xiaoke | 2022 | Chinese Journal of Traditional Chinese Medicine | 1 | 1 | National project | RCT | To explore the effects of acupuncture on the biological characteristics, sex hormone levels, glucose and lipid metabolism levels and obstetric outcomes of patients with infertility syndrome of kidney deficiency and liver depression, so as to provide clinical objective basis for acupuncture treatment of this syndrome. | 392 | Polycystic ovary syndrome |  |
| 22 | Wei Huijun; Li Jinxia; Gao Xuejuan; Yin Yanru; Wang Mengchang | 2022 | Shanghai Journal of Acupuncture | 1 | 1 | Provincial-level project | RCT | To observe the effect of acupuncture in accordance with menstrual cycle staging on immune regulation and clinical pregnancy rate of infertility patients with kidney deficiency polycystic ovary syndrome (PCOS). | 100 | Polycystic ovary syndrome |  |
| 23 | Huang Weimei; Xia Dongbin; Wang Xilong; Lin Haochun; Ouyang Huailiang | 2022 | Asia-pacific traditional medicine | 1 | 1 | None | RCT | To explore the effect of acupoint embedding guided by the theory of strengthening spleen and expelling phlegm on the level of Omentin-1 in patients with polycystic ovary syndrome (PCOS). | 108 | Polycystic ovary syndrome |  |
| 24 | Weizhen juice; Zhang Dandan; Song Haifeng; He Xiumei | 2022 | Journal of Shanxi Health Vocational College | 1 | 1 | None | RCT | To study the clinical effect of warm acupuncture and moxibustion combined with clomiphene (CC) and progesterone on infertility patients with kidney deficiency polycystic ovary syndrome (PCOS). | 110 | Polycystic ovary syndrome |  |
| 25 | Li Yimei; Zhou Tao; Zhang Tianhui; Zhao Xiaojun | 2022 | Marriage, childbearing and health | 1 | 1 | None | RCT | To investigate the effect of intradermal acupuncture on uterus, ovary and other acupoints to improve hormone levels, promote ovulation, restore menstruation and increase pregnancy rate | 20 | Ovulation disturbance |  |
| 26 | Li Aiyu | 2022 | Health friend | 1 | 1 | None | RCT | To investigate the application of fire acupuncture combined with ear point compression bean in polycystic ovary syndrome (PCOS) patients with multiple follicles and its effect on pregnancy rate | 92 | Polycystic ovary syndrome |  |
| 27 | Shan Xingyu; Lai Shuting | 2022 | Practical clinical integration of traditional Chinese and Western medicine | 1 | 1 | None | RCT | Objective: To explore the effect of acupuncture and moxibustion on infertility caused by kidney-yang deficiency polycystic ovary syndrome (PCOS) | 60 | Polycystic ovary syndrome |  |
| 28 | Li Yonghong; Ke Yan; Liu Yujie; Feng Ting; Li Yuchang; Li Hui; Zhang Wen | 2022 | Journal of Liaoning University of Chinese Medicine | 1 | 1 | Municipal subject | RCT | To explore the therapeutic effect of Tiaoretongdu acupuncture combined with letrozole on infertility caused by kidney-yang deficiency polycystic ovary syndrome (PCOS) and its influence on lipid metabolism and sex hormone levels | 124 | Polycystic ovary syndrome |  |
| 29 | Zhang Xiaoxue; Guo Xinfeng; Wang Hanlin; Xie Changcai | 2022 | Modern TCM clinic | 1 | 1 | Provincial-level project | RCT | To observe the efficacy and safety of regulating injection in the treatment of ovulation disorder in polycystic ovary syndrome (PCOS). | 65 | Polycystic ovary syndrome |  |
| 30 | Li Li; Wang Ying; Liu Hui | 2022 | Guangming Chinese Medicine | 1 | 1 | None | RCT | To investigate the clinical effect of acupuncture on luteinization syndrome of unruptured follicles (LUFS) | 72 | Luteinization syndrome of unruptured follicles |  |
| 31 | Zhu Lijuan; Xiao Shaofang; Zhang Minxia | 2022 | Journal of Jiangxi University of Chinese Medicine | 1 | 1 | Provincial-level project | RCT | To observe the clinical effect of Dain-35 combined acupoint embedding on infertility of polycystic ovary syndrome (PCOS) | 60 | Polycystic ovary syndrome |  |
| 32 | Li Sichen | 2022 | Changchun University of Chinese Medicine | 2 | 1 | None | RCT | It is necessary to take the ovulation disorder infertility patients with liver depression and kidney deficiency as the clinical research target, and make a comparison between the intervention of ovulation with qi stimulating injection and chorionic gonadotropin used in clinical practice to objectively evaluate the clinical effectiveness of qi stimulating injection and explore the degree of improvement of ovulation in patients with qi stimulating injection | 72 | Ovulation disturbance |  |
| 33 | Yin Guochao, Yang Junwei, Chen Nianhui, ZHANG Yide, QIN Guiqiong, DUAN Jinliang | 2021 | Journal of Translational medicine | 1 | 1 | Provincial and ministerial projects | RCT | To observe the effect of electroacupuncture (EA) combined therapy on endometrial receptivity in patients with polycystic ovary syndrome (PCOS) | 70 | Polycystic ovary syndrome |  |
| 34 | Y. Zhang, Lan, Y.Y. | 2021 | Chinese Medicine Modern Distance Education of China | 1 | 1 | Provincial and ministerial project | RCT | To observe the curative effect of Zhuang medicine shallow pricking therapy in the treatment of anovulatory infertility. | 62 | Ovulation inhibition |  |
| 35 | Li, M. Z. | 2021 | Chinese Baby | 1 | 1 | None | RCT | Analysis of the effect of letrozole combined with cupping and weight reduction for ovulation and pregnancy rate in polycystic ovary syndrome infertility. | 88 | polycystic ovarian syndrome, PCOS |  |
| 36 | Z. F. Zhang | 2021 | Smart Healthcare | 1 | 1 | None | RCT | To analyze the effect of warm acupuncture on endometrial thickness and pregnancy  outcome | 60 | Thin endometrium |  |
| 37 | Y. N. Zhang, Zhao, J.H., Wang, L.L., An, L.P. | 2021 | Our Health | 1 | 1 | None | RCT | To observe the ovulation rate and pregnancy rate of moxibustion at Yinlian (LI 20) combined with letrozole in the ovulation induction treatment for ovulatory dysfunction infertility | 60 | Ovulation failure infertility |  |
| 38 | Q. P. Lin, Liu, Y., Xu, J.B., Yang, J., You, X.M., Zhang, J.X. | 2021 | World Journal of Acupuncture-Moxibustion | 1 | 2 | Provincial and ministerial project | RCT | observe the effect of the combined therapy of electroacupuncture (EA) and ginger-isolated moxibustion on endometrial receptivity in patients with polycystic ovarian syndrome (PCOS). | 70 | polycystic ovarian syndrome, PCOS |  |
| 39 | Y. R. Liang, Li, K.Y., Zeng, J.M. | 2021 | Journal of Practical Traditional Chinese Medicine | 1 | 1 | None | RCT | To observe the effect of Huolong jar Therapy in complementary treatment of ovulatory dysfunction infertility with kidney Yang deficiency | 60 | ovulatory dysfunction |  |
| 40 | Y. P. Yang | 2021 | Shanghai Journal of Acupuncture and Moxibustion | 1 | 1 | Provincial and ministerial project | RCT | To observe the meridian regulating and pregnancy promoting effects of fire needle on infertility with kidney deficiency and blood stasis and its clinical efficacy | 94 | Not reported |  |
| 41 | Y. M. Wang | 2021 | Shanxi University of Chinese Medicine | 2 | 1 | None | RCT | To observe the effect of acupoint catgut embedding therapy on the endometrial receptivity during ovulation induction period in PCOS infertility patients with kidney deficiency and blood stasis syndrome | 80 | polycystic ovarian syndrome, PCOS |  |
| 42 | Z. Y. Lin, Liao, Q.L. | 2021 | Journal of External Therapy of Traditional Chinese Medicine | 1 | 1 | Provincial and ministerial project | RCT | To observe the clinical effect of acupoint catgut embedding in the treatment of polycystic ovary syndrome (PCOS). | 90 | polycystic ovarian syndrome, PCOS |  |
| 43 | Z. F. Mei | 2021 | Anhui University of Traditional Chinese Medicine | 2 | 1 | None | RCT | To observe the clinical efficacy of acupuncture combined with levocarnitine oral liquid in the treatment of asthenospermia with kidney yang deficiency. | 70 |  | Idiopathic male infertility |
| 44 | Z. Z. Meng | 2021 | Liaoning Journal of Traditional Chinese Medicine | 1 | 1 | None | RCT | To observe the effect of acupuncture assisted hydrotubation in treatment of women with tubal infertility. | 60 | tubal infertility. |  |
| 45 | C. H. Zhang, Wang, H.Y., Bi, Y.H., Chen, G.Z., Gao, X.Y. | 2021 | Journal of Clinical Acupuncture and Moxibustion | 1 | 1 | Provincial and ministerial project | RCT | To investigate the effect of BO's abdominal acupuncture combined with human chorionic gonadotropin (HCG) on serum Estradiol(E2), Luteinizing hormone(LH), follicle-stimulating hormone (FSH) and ovulation rate in patients with luteinized uNot reporteduptured follicle syndrome (FSH) and ovulation rate in patients with luteinized uNot reporteduptured follicle syndrome | 90 | luteinized uNot reporteduptured follicle syndrome,LUFS |  |
| 46 | Y. Y. Yang | 2021 | Modern Medicine and Health Research Electronic Journal | 1 | 1 | None | RCT | To explore the effect of acupuncture method of transfer supervision on pregnancy and serum follicle-stimulating hormone (FSH), luteinizing hormone (LH), estradiol (E2), and testosterone (T) levels in infertility patients with polycystic ovary syndrome. | 115 | polycystic ovarian syndrome, PCOS |  |
| 47 | H. J. Zhang, Huang, B.Z., Liang, X. | 2021 | Doctor | 1 | 1 | None | RCT | To explore the effect of acupoint acupuncture on improving pregnancy outcomes and sex hormone levels of infertile patients with ovulation disorders | 48 | ovulatory dysfunction |  |
| 48 | Y. J. Wang, , Ran, J., Fang, Y.L | 2021 | Internal Medicine | 1 | 1 | None | RCT | To investigate the clinical effect of acupuncture therapy for regulating menstruation and promoting pregnancy in the treatment of patients with decreased ovarian reserve function (DOR). | 60 | decreased ovarian reserve function (DOR). |  |
| 49 | C. M. Yu | 2021 | Pharmacy Weekly | 1 | 1 | None | RCT | To investigate the clinical effects of acupuncture combined with western medicine on endometrial thickness, ovulation rate and pregnancy in patients with polycystic ovary syndrome (PCOS). | 79 | polycystic ovary syndrome |  |
| 50 | J. Wu | 2020 | Clinical Journal of Chinese Medicine | 1 | 1 | None | RCT | Analysis of the effectiveness of acupuncture in the treatment of serum antisperm antibody-positive infertility. | 90 | Infertility with positive serum antisperm antibodies |  |
| 51 | W. W. Jia, C., Yin, Y. | 2020 | Journal of External Therapy of Traditional Chinese Medicine | 1 | 1 | None | RCT | Observation of the effects of "Ren-Du" moxibustion with ginger on sperm quality and anxiety in hypospermia patients with kidney yang. deficiency | 40 |  | Idiopathic male infertility |
| 52 | S. R. Liang, Huang, X.T., He, D.J., Yang, Y.Q., Xiang, X., Zhang, X.Y. | 2020 | Lishizhen Medicine and Materia Medica Research | 1 | 1 | Provincial and ministerial project | RCT | Observation on the clinical efficacy of regulating menstruation acupuncture combined with tuoxibustion in the treatment of infertility caused by luteinization syndrome of uNot reporteduptured follicle | 72 | luteinized uNot reporteduptured follicle syndrome,LUFS |  |
| 53 | X. Xiang, Huang, X.T., Ge, M.,Gao, J. | 2020 | Hebei Journal of Traditional Chinese Medicine | 1 | 1 | Provincial and ministerial project | RCT | To observe the effect of acupuncture and moxibustion at the Eight Points on perifollicular blood flow parameters and pregnancy rate in primary ovarian insufficiency (POI) infertility in the occult phase. | 75 | ovarian insufficiency |  |
| 54 | X. Y. Peng | 2020 | Chinese Community Doctors | 1 | 1 | None | RCT | To observe the clinical effect of acupuncture combined with metformin in the treatment of polycystic ovary infertility | 60 | polycystic ovarian syndrome, PCOS |  |
| 55 | M. L. Zhang, Wang, X.G., Shan, Z.P., Dong, B.Q. | 2020 | Shanghai Journal of Acupuncture and Moxibustion | 1 | 1 | Provincial and ministerial project | RCT | To observe the clinical efficacy of acupuncture combined with metformin in the treatment of polycystic ovary infertility. | 64 | polycystic ovarian syndrome, PCOS |  |
| 56 | L. P. Yang, Chen, Y., Chen, Y.J. | 2020 | Shanghai Journal of Acupuncture and Moxibustion | 1 | 1 | Provincial and ministerial project | RCT | Observing the clinical efficacy and pregnancy outcome of acupuncture in the treatment of luteal insufficiency | 60 | luteal phase defect |  |
| 57 | B. L. Wang | 2020 | Fujian University of Traditional Chinese Medicine | 2 | 1 | None | RCT | To observe the clinical efficacy of acupuncture cycle therapy in the treatment of ovulation disorders in PCOS patients with kidney deficiency and blood stasis | 68 | ovulation inhibition |  |
| 58 | H. Mi | 2020 | Fashion Baby | 1 | 1 | None | RCT | Exploring the clinical effectiveness of acupuncture treatment in female ovulatory dysfunctional infertility | 64 | ovulation inhibition |  |
| 59 | C. X. Xu, Mu, Y.Y. | 2020 | China Journal of Traditional Chinese Medicine and Pharmacy | 1 | 1 | None | RCT | To investigate the efficacy of Dumaiwen acupuncture combined with clomiphene on infertility caused by polycystic ovary syndrome | 82 | polycystic ovarian syndrome, PCOS |  |
| 60 | L. F. Chen | 2020 | Contemporary Medical Symposium | 1 | 1 | None | RCT | To investigate the effect of ethinylestradiol cyproterone tablets combined with acupuncture therapy on infertility of polycystic ovary syndrome | 86 | polycystic ovarian syndrome, PCOS |  |
| 61 | J. Y. Wang, Ma, Y., Du, W.N., Wang, B.C., Wu, L.Q. | 2020 | China Modern Medicine | 1 | 1 | Provincial and ministerial project | RCT | To investigate the effect of metformin combined with acupuncture on glucolipid metabolism and adipokine of obese PCOS patients | 90 | polycystic ovarian syndrome, PCOS |  |
| 62 | T. L. Ye, H. X. Cheng | 2020 | Journal of Acupuncture and Tuina Science: Acupuncture, Moxibustion and Therapeutic Massage | 1 | 2 | None | RCT | To explore the effect and related mechanism of heat-sensitive moxibustion plus Clomifene citrate capsules for infertility due to polycystic ovary syndrome (PCOS). | 70 | polycystic ovarian syndrome, PCOS |  |
| 63 | S.Z. zheng | 2020 | Lishizhen Medicine and Materia Medica Research | 1 | 1 | Provincial and ministerial project | RCT | To explore the mechanism of interventional acupuncture in CC cycle ovulatory infertility | 66 | ovulation inhibition |  |
| 64 | M. Jiang | 2020 | Reflexology and Rehabilitation Medicine | 1 | 1 | None | RCT | To explore the effect of moxibustion in treating polycystic ovary syndrome complicated with infertility | 88 | polycystic ovarian syndrome, PCOS |  |
| 65 | Z. G. Shao | 2020 | China Health Care & Nutrition | 1 | 1 | None | RCT | To explore the effect of warm needle combined with gourd moxibustion on thin endometrium | 30 | thin endometrium |  |
| 66 | Q. X. Zhuang, Chen, Q.N., Wu, K.Z., Zhang, J.N., Liao,Q.C. | 2020 | Guangming Journal of Chinese Medicine | 1 | 1 | Provincial and ministerial project | RCT | To observe the ovulation laboratory indexes and pregnancy rate of patients with recurrent luteinized infertility with uNot reporteduptured follicles by acupoint moxibustion. | 84 | luteinized uNot reporteduptured follicle syndrome |  |
| 67 | X. X. Wei | 2019 | Anhui University of Traditional Chinese Medicine | 2 | 1 | None | RCT | Evaluating the clinical value of acupuncture combined with acupressure, as well as providing a simple and inexpensive combined Chinese and Western medicine treatment option for patients with tubal obstruction infertility | 60 | tubal obstruction |  |
| 68 | M. Q. Zhou | 2019 | Chengdu University of TCM | 2 | 1 | None | RCT | To observe the difference in clinical efficacy between western medicine and western medicine combined with acupuncture in patients with polycystic ovary syndrome infertility, and to explore the effect of acupuncture on pregnancy outcome | 64 | polycystic ovarian syndrome, PCOS |  |
| 69 | X. S. Xu | 2019 | Shandong University of Traditional Chinese Medicine | 2 | 1 | None | RCT | Finding the superiority between the crossmoxibustion,Baliao moxibustioncombind with femostone and pure femostone to curediminished ovarian reserve. | 40 | diminished ovarian reserve |  |
| 70 | L. X.Chen | 2019 | Fujian University of Traditional Chinese Medicine | 2 | 1 | None | RCT | To analyse the effect of acupuncture combined with heat-sensitive moxibustion in the treatment of luteinization syndrome of uNot reporteduptured follicle of "stasis uterine" type | 60 | luteinized uNot reporteduptured follicle syndrome,LUFS |  |
| 71 | Z. S. Guo | 2019 | Practical Clinical Journal of Integrated Traditional Chinese and Western Medicine | 1 | 1 | None | RCT | To analyse the effect of Oral ethinylestradiol cyproterone tablets combined with acupuncture in the treatment of infertility with polycystic ovary syndrome | 92 | polycystic ovarian syndrome, PCOS |  |
| 72 | L.Chen | 2019 | China Practical Medicine | 1 | 1 | None | RCT | To analyse the effect of acupoint catgut embedding in the treatment of clomiphene resistant polycystic ovary syndrome | 68 | polycystic ovarian syndrome, PCOS |  |
| 73 | Q. X. Lang, Mo, J. | 2019 | World Latest Medicine Information | 1 | 1 | None | RCT | To analyse the effect of warm acupuncture on thin endometrial infertility | 92 | thin endometrium |  |
| 74 | K. Xie, Wang, L.L., | 2019 | Chinese Journal of General Practice | 1 | 1 | National-level fund projects | RCT | To analyze the effects of acupuncture and moxibustion on the levels of sex hormones, insulin (INS), cortisol (Cor), 17- hydroxyprogesterone (17-OHP), dehydroepiandrosterone sulfate (DHEA-S), pregnancy rate, ovulation rate and abortion rate. | 100 | polycystic ovarian syndrome, PCOS |  |
| 75 | X. Y. Zhang, Ying, J., Deng, H.P., Cheng, K., Wag, Q., Pan, W., Ji, J., Wang, K.X., Zhao, L. | 2019 | Journal of Clinical Acupuncture and Moxibustion | 1 | 1 | None | RCT | To observe the correlation between the clinical efficacy of moxibustion on CV8 and CV4 treating male infertility and the two acupoints temperature． | 102 |  | Idiopathic male infertility |
| 76 | W. T. Gai | 2019 | Heilongjiang University of Chinese Medicine | 2 | 1 | None | RCT | To observe the clinical efficacy, body mass index (BMI) and safety of venesection and cupping combined with clomiphene in the treatment of PCOS infertility, in order to provide an effective new clinical treatment for PCOS infertility patients | 72 | polycystic ovarian syndrome, PCOS |  |
| 77 | M. Q. Zheng, Weng, C. | 2019 | Asia-Pacific Traditional Medicine | 1 | 1 | None | RCT | To observe the clinical efficacy of clomiphene combined with Mingmen Bazhen Point in the treatment of infertility in polycystic ovary syndrome with kidney-Yang deficiency | 64 | polycystic ovarian syndrome, PCOS |  |
| 78 | J. Meng | 2019 | Henan Traditional Chinese Medicine | 1 | 1 | Provincial and ministerial project | RCT | To observe the clinical efficacy of Meihua needle treatment in uNot reporteduptured follicular luteinization syndrome of kidney deficiency and blood stasis | 66 | luteinized uNot reporteduptured follicle syndrome,LUFS |  |
| 79 | Y. Li, Chen, H.F., Zeng, W., Luo, J., Liu, Y. | 2019 | Clinical Journal of Chinese Medicine | 1 | 1 | None | RCT | To observe the clinical efficacy of suspension moxibustion plus aspirin for thin endometrial repair | 80 | luteinized uNot reporteduptured follicle syndrome,LUFS |  |
| 80 | Y. H. Zhang | 2019 | Shanghai Journal of Acupuncture and Moxibustion | 1 | 1 | None | RCT | To observe the effect of Tongyuan acupuncture on the pregnancy success rate of patients with ovulatory infertility. | 106 | ovulation inhibition |  |
| 81 | L. L. Ji, Shen, J., Luo, Y., Jiang, Y.W., Ai, B.W. | 2019 | Jiangsu Journal of Traditional Chinese Medicine | 1 | 1 | None | RCT | To observe the clinical efficacy of catgut embedding at acupoint on infertility caused by follicular dysplasia. | 60 | Follicular Maldevelopment,FM |  |
| 82 | C. P. Yan, Fan, C., Yan, H., Zhang, L.X., Zhong, Y.F., Zhou, X.X. | 2019 | Journal of New Chinese Medicine | 1 | 1 | None | RCT | To observe the clinical efficacy of acupuncture with clomiphene ovulation promotion on follicular development and endometrium in patients with polycystic ovary syndrome (PCOS) with ovulatory disorders. | 60 | polycystic ovarian syndrome, PCOS |  |
| 83 | S. L. Hou, Zhao, P., Shi, Y.L. | 2019 | Shanghai Journal of Acupuncture and Moxibustion | 1 | 1 | None | RCT | To observe the clinical efficacy of acupuncture combined with Western medicine in treating infertility of premature ovarian failure | 96 | prmature pvarian failure,POF |  |
| 84 | G. Q. Yang | 2019 | Clinical Journal of Chinese Medicine | 1 | 1 | None | RCT | To explore the effect of acupuncture on infertility from endocrine disorders, and compare with clomiphene | 80 | endocrine disorders |  |
| 85 | L. Wang, Wang, L., Wang, X.R. | 2019 | Clinical Journal of Diabetes World 2019 | 1 | 1 | None | RCT | To discuss the clinical efficacy and safety of moxibustion combined with western medicine in treating infertility caused by obese polycystic ovary syndrome. | 72 | polycystic ovarian syndrome, PCOS |  |
| 86 | L. Yu, L. Cao, J. Xie and Y. Shi | 2018 | Chinese Acupuncture & Moxibustion | 1 | 1 | Provincial and ministerial project | RCT | To compare the difference in the therapeutic effects on ovulation disorder in polycystic ovary syndrome (PCOS) between the combined therapy of electroacupuncture and clomiphene and the single medication of clomiphene and to explore the effect mechanism. | 80 | polycystic ovarian syndrome, PCOS |  |
| 87 | L. Q. Yu, CAO, L.Y., Xie, Q., Shi, Y. | 2018 | Chinese Acupuncture & Moxibustion | 1 | 2 | Provincial and ministerial project | RCT | To observe the clinical efficacy of electroacupuncture combined with clomiphene on ovulation promotion and pregnancy promotion in polycystic ovary syndrome | 80 | polycystic ovarian syndrome, PCOS |  |
| 88 | S. C. Peng | 2018 | Guangzhou University of Chinese Medicine | 2 | 1 | None | RCT | To observe clinical curative effect of Acupuncture combined with Letroz01e(LE)+Gonadotropin(Gn)on Infertile Women with Polycystic Ovary Syndrome(PCOS)insensitive to Clomiphene | 68 | polycystic ovarian syndrome, PCOS |  |
| 89 | Z. R. Yang | 2018 | Yunnan University of Traditional Chinese Medicine | 2 | 1 | None | RCT | To observe the effect of acupuncture using Daoqi manipulation and different therapy in different time on tubal infertilty | 72 | tubal obstruction |  |
| 90 | L. N. Shan | 2018 | Guangxi University of Chinese Medicine | 2 | 1 | None | RCT | Through the rigorous and scientific clinical research, to survey and assess the comprehensive therapeutic c effect of e1 electroacupuncture on infertility with polycystic ovary syndrome caused by kidney deficiency and liver depression and the impact on uterine artery hemodynamics． | 79 | polycystic ovarian syndrome, PCOS |  |
| 91 | J. Xu and Y. Zuo | 2018 | Chinese Acupuncture & Moxibustion | 1 | 1 | Provincial and ministerial project | RCT | To observe the efficacy differences between acupuncture combined with medication and medication alone for infertility patients with polycystic ovary syndrome (PCOS). | 60 | polycystic ovarian syndrome, PCOS |  |
| 92 | J. Xu and Y. Zuo | 2018 | Chinese Acupuncture & Moxibustion | 1 | 2 | Provincial and ministerial project | RCT | To observe the efficacy differences between acupuncture combined with medication and medication alone for infertility patients with polycystic ovary syndrome (PCOS). | 60 | polycystic ovarian syndrome, PCOS |  |
| 93 | Y. D. Yuan, Yan, Y.T., Wei, R.H., Shen, D. | 2018 | Shanghai Journal of Acupuncture and Moxibustion | 1 | 1 | None | RCT | To observe the therapeutic efficacy of acupuncture-moxibustion plus sequential low—dose human menopausal gonadotropin(hMG)in the treatment of infertility in clomiphene citrate(CC)一resistant polycystic ovary syndrome(PCOS)． | 57 | polycystic ovarian syndrome, PCOS |  |
| 94 | L. R. Chen, Li, J., Pan, X.X., Xie, S.Y., Huang, C.R. | 2018 | Hunan Journal of Traditional Chinese Medicine | 1 | 1 | Provincial and ministerial project | RCT | To observe the therapeutic efficacy of Zhu Lian acupuncture combined with Wen Ren Regulation and supervision method in the treatment of ovulatory dysfunction infertility | 70 | UNot reportedeported |  |
| 95 | Y. Chen | 2018 | Journal of Yunnan University of Traditional Chinese Medicine | 1 | 1 | Provincial and ministerial project | RCT | To evaluate the clinical efficacy of thunder moxibustion combined with ovulation monitoring in the treatment of adenomyosis complicated with infertility. | 60 | adenomyosis,AM |  |
| 96 | Y. Q. Niu, Tian, C., Li, J., Liu, H., Chang, X.L. | 2018 | Practical Clinical Medicine | 1 | 1 | Provincial and ministerial project | RCT | To observe the therapeutic efficacy of prednisolone acetate combined with acupuncture in the treatment of unexplained infertility | 55 | Unexplained infertility |  |
| 97 | X. Qiu, Xu, M.Y. | 2018 | HOSPITAL AND MEDICISE | 1 | 1 | None | RCT | To explore the efficiency of acupuncture and moxibustion in infertility patients with polycystic ovary syndrome | 75 | polycystic ovarian syndrome, PCOS |  |
| 98 | J. L. Luo, Li, Y.M., Zheng, S.Z. | 2018 | Jilin Journal of Chinese Medicine | 1 | 1 | Provincial and ministerial project | RCT | To explore the efficiency of tong Yuan acupuncture combined with drugs in the treatment of ovulatory dysfunction infertility | 56 | ovulatory dysfunction infertility |  |
| 99 | X. M. You, Yang, J.,Xu, J.B., Liu, Y., Huang, J. Liu, Q.P.,Zhang, J.X., Dun, J.J., Huang, S. | 2018 | World Journal of Acupuncture-Moxibustion | 1 | 1 | National-level fund projects | RCT | To explore the effect of warm acupuncture on endometrial thickness and pregnancy outcome. | 40 | thin endometrium |  |
| 100 | Y. Li, Chen, H.F., Zeng, W., Luo, J., Liu, Y. | 2018 | Clinical Journal of Chinese Medicine | 1 | 1 | Provincial and ministerial project | RCT | To explore the efficiency of suspension moxibustion plus aspirin for thin endometrial repair | 120 | thin endometrium |  |
| 101 | L. Yang | 2018 | Health Protection and Promotion | 1 | 1 | None | RCT | To explore the clinical effect and safety of acupuncture therapy for infertility. | 60 | ovulation inhibition |  |
| 102 | Y. E. Liu | 2018 | Electronic Journal of Practical Gynecologic Endocrinology | 1 | 1 | None | RCT | To explore the clinical effect of acupuncture and moxibustion in treatment of ovulatory dyssterility and its influence on abortion | 52 | ovulation inhibition |  |
| 103 | Y. Wang, Zheng, H.Y. | 2018 | Shenzhen Journal of Integrated Traditional Chinese and Western Medicine | 1 | 1 | None | RCT | To explore the feasibility of electroacupuncture in the treatment of ovulation disorder of polycystic ovary syndrome. | 68 | polycystic ovarian syndrome, PCOS |  |
| 104 | Y. L. Fang | 2018 | Zhejiang Chinese Medical University | 2 | 1 | None | RCT | To study the effects of acupuncture combined with auricular points on ovulation rate, pregnancy rate, recurrence rate of LUF cycle and high temperature phase (HPS) score of basal body temperature (BBT) in patients with uNot reporteduptured luteinized follicle syndrome (LUFS) of kidney deficiency and blood stasis. | 72 | luteinized uNot reporteduptured follicle syndrome,LUFS |  |
| 105 | N. Li | 2017 | Guide of China Medicine | 1 | 1 | None | RCT | Analysis and discussion on the clinical effect of acupoint moxibustion on infertility. | 60 | UNot reportedeported |  |
| 106 | X. P. Huang, Li, G.H. | 2017 | Inner Mongolia Journal of Traditional Chinese | 1 | 1 | National-level fund projects | RCT | To observe the therapeutic efficacy of acupuncture on ovulatory dysfunction infertility | 87 | ovulation inhibition |  |
| 107 | Y. Yin, Zhang, Y.P., Zhao, P.C., Guo, G.R. | 2017 | Jiangsu Journal of Traditional Chinese Medicine | 1 | 1 | Provincial and ministerial project | RCT | To observe the therapeutic efficacy of modified acupoint catgut embedding combined with letrozole on ovulation induction and pregnancy in PCOS infertile patients | 110 | polycystic ovarian syndrome, PCOS |  |
| 108 | Y. T. Chen, Li, Y.M.,Luo, J.L., Chen, Y.J., Zheng, S.J. | 2017 | Shanghai Journal of Acupuncture and Moxibustion | 1 | 1 | National-level fund projects | RCT | To observe the therapeutic efficacy of tongyuan acupuncture combined with drugs in the treatment of refractory polycystic ovary syndrome | 60 | polycystic ovarian syndrome, PCOS |  |
| 109 | X. F. Dai | 2017 | Journal of New Chinese Medicine | 1 | 1 | None | RCT | To observe the efficiency of the treatment for infertility with obesity polycystic ovary syndrome by acupuncture and auricular sticking combined with metformin | 98 | polycystic ovarian syndrome, PCOS |  |
| 110 | F. H. Yang, Qing, W.H. | 2017 | Inner Mongolia Journal of Traditional Chinese | 1 | 1 | Provincial and ministerial project | RCT | To observe the efficiency of acupuncture combined with auricular point pressing in treatment of infertility in LUFS | 64 | luteinized uNot reporteduptured follicle syndrome,LUFS |  |
| 111 | J. J. Zhu | 2017 | Guangzhou University of Chinese Medicine | 2 | 1 | None | RCT | To observe the efficiency of electric acupuncture at Navel Thursday point plus wheat grain moxibustion | 60 | ovulation inhibition |  |
| 112 | Y. Zhu, Cao, Q. An, C.P., Chang, C.F. | 2017 | Shanghai Journal of Acupuncture and Moxibustion | 1 | 1 | Provincial and ministerial project | RCT | To observe the clinical efficacy and safety of acupuncture combined with medicine in treating infertility caused by polycystic ovary syndrome. | 90 | polycystic ovarian syndrome, PCOS |  |
| 113 | J. Li, Chen, L.R., Huang, C.R., Pan, X.T., Xie, Y.S. | 2017 | Shanghai Journal of Acupuncture and Moxibustion | 1 | 1 | Provincial and ministerial project | RCT | To observe the efficacy difference between ZHU Lian’s acupuncture plus Wen Ren Tiao Shu method (warming the Conception Vessel and regulating back-Shu points) and ovulation drugs in treating anovulatory infertility. | 60 | ovulation inhibition |  |
| 114 | C. X. Li, Di, W.J. | 2017 | World Latest Medicine Information | 1 | 1 | None | RCT | To observe the clinical efficacy of acupuncture in the treatment of uterine cold infertility | 40 | UNot reportedeported |  |
| 115 | Y. X. Song, Li, Y.K. | 2017 | Shaanxi Journal of Traditional Chinese Medicine | 1 | 1 | None | RCT | To explore the application value of warm acupuncture combined with cycle therapy in the treatment of infertility caused by polycystic ovary syndrome (POCS) with phlegm-dampness complicated with infertility. | 90 | polycystic ovarian syndrome, PCOS |  |
| 116 | L.Yang | 2017 | Health Protection and Promotion | 1 | 1 | none | RCT | Exploring the clinical effectiveness and safety of acupuncture therapy in the treatment of infertility | 120 | Not reported |  |
| 117 | Huang.X.S | 2017 | Shaanxi Journal of Traditional Chinese Medicine | 1 | 1 | none | RCT | Exploring the efficacy of acupuncture-based treatment for infertility in obese polycystic ovary syndrome | 100 | Polycystic Ovary Syndrome |  |
| 118 | J.Tang | 2017 | Zhejiang Journal of Traditional Chinese Medicine | 1 | 1 | none | RCT | Not reported | 104 | Polycystic Ovary Syndrome |  |
| 119 | Liu.J.H;He.F.E;Wen.L.F;He.Z.Y;Li.J.J | 2017 | Northern Medicine | 1 | 1 | none | RCT | To summarize and analyze the clinical effects of treatment of oligozoospermia using levocannabinoid combined with acupuncture. | 120 |  | Idiopathic Male Infertility |
| 120 | Su.B.Y | 2016 | Guangzhou University of Chinese Medicine | 2 | 1 | none | RCT | To observe the clinical efficacy of abdominal acupuncture combined with moxa box moxibustion in the treatment of kidney deficiency and cold infertility, in order to broaden the efficacy of abdominal acupuncture and the scope of application of acupuncture combined with moxibustion in the treatment of infertility, and to provide rigorous scientific research data and new clinical ideas for clinical abdominal acupuncture combined with moxibustion in the treatment of infertility. | 66 | Ovulation Disorder |  |
| 121 | Zhuo.Y.Y;Wu.J.M;Lin.W.S;M.Pi;Chen.P.D;Yang.Z.X | 2016 | Chinese Acupuncture & Moxibustion | 1 | 1 | Provincial and ministerial projects | RCT | To compare the clinical efficacy of the "Regulating Ren and Tongdu Acupuncture Method" with that of oral clomiphene in the treatment of polycystic ovary syndrome infertility. | 100 | Polycystic Ovary Syndrome |  |
| 122 | Zhuo, Y; Wu, J; Lin, W; Pi, M; Chen, P; Yang, Z | 2016 | Chinese Acupuncture & Moxibustion | 1 | 1 | Provincial and ministerial projects | RCT | To compare the clinical efficacy of the "Tong Ren Tong Du" acupuncture method with that of oral clomiphene in the treatment of polycystic ovary syndrome (PCOS) infertility. | 100 | Polycystic Ovary Syndrome |  |
| 123 | Wei.H.Y | 2016 | Zhejiang Clinical Medical Journal | 1 | 1 | none | RCT | To observe the efficacy of letrozole combined with acupuncture in the treatment of ovulation in infertile patients with polycystic ovary syndrome. | 78 | Polycystic Ovary Syndrome |  |
| 124 | L.Huang | 2016 | Guangzhou University of Chinese Medicine | 2 | 1 | none | RCT | To observe the clinical efficacy of TongYuan Acupuncture in the treatment of polycystic ovary syndrome combined with infertility, to evaluate the clinical effectiveness of TongYuan Acupuncture, and to explore the mechanism of TongYuan Acupuncture, in order to provide clinical reference for proving the advantages of TongYuan Acupuncture and further multi-centre and large sample clinical studies. | 60 | Polycystic Ovary Syndrome |  |
| 125 | X.Sun;Qi.Q.H;Kong.S.F;Zhong.J.Y | 2016 | Chinese Journal of Information on Traditional Chinese Medicine | 1 | 1 | Provincial and ministerial projects | RCT | To observe the clinical efficacy of western medicine combined with acupuncture in the treatment of infertility in polycystic ovary syndrome. | 120 | Polycystic Ovary Syndrome |  |
| 126 | Tang.L.M | 2016 | Shanxi Journal of Traditional Chinese Medicine | 1 | 1 | none | RCT | To observe the clinical effects of sequential acupuncture therapy in the treatment of tubal obstructive infertility. | 60 | Obstruction of Fallopian Tube |  |
| 127 | Zhai.W.P | 2016 | Guangzhou University of Chinese Medicine | 2 | 1 | none | RCT | To observe the clinical efficacy of Yi Qi ovulation method combined with clomiphene in the treatment of ovulation disorders in polycystic ovary syndrome (PCOS). | 60 | Polycystic Ovary Syndrome |  |
| 128 | Fang,Q,X;P.Zou;Li.K.Y | 2016 | Beijing Journal of Traditional Chinese Medicine | 1 | 1 | Provincial and ministerial projects | RCT | To observe the effect of acupuncture for ovulation on follicular development and reproductive hormones in patients with polycystic ovary syndrome and to evaluate its clinical efficacy. | 60 | Polycystic Ovary Syndrome |  |
| 129 | Wang.W.Y;Ni.L.W;Q.Geng | 2016 | Lishizhen Medicine and Materia Medica Research | 1 | 1 | National project | RCT | To observe the clinical efficacy of acupuncture in the treatment of oxidative stress injury in oligo- and hypospermia. | 80 |  | Idiopathic Male Infertility |
| 130 | Wang.Y.G | 2016 | Guangdong Trace Elements Science | 1 | 1 | none | RCT | Observation and investigation on the clinical efficacy of acupuncture in the treatment of polycystic ovary syndrome causing infertility | 66 | Polycystic Ovary Syndrome |  |
| 131 | Zhang.Z.S;Qiao.Y.J | 2016 | Journal of External Therapy of Traditional Chinese Medicine | 1 | 1 | none | RCT | A study on the improvement and clinical efficacy of milli-fire acupuncture combined with general acupuncture on sperm motility in weak spermatozoa with deficiency of kidney yang. | 64 |  | Idiopathic Male Infertility |
| 132 | Lv.X.W | 2016 | Inner Mongolia Journal of Traditional Chinese Medicine | 1 | 1 | none | RCT | To study the clinical effects of acupuncture in the treatment of polycystic ovary syndrome combined with infertility. | 200 | Polycystic Ovary Syndrome |  |
| 133 | Liu.L.Y | 2015 | Guangxi University of Chinese Medicine | 2 | 1 | none | RCT | The effectiveness, safety and feasibility of acupuncture combined with human chorionic gonadotropin (HCG) in the treatment of luteinization syndrome of uNot reporteduptured follicles (LUFS) were objectively evaluated to provide a basis for future treatment protocols and technical specifications for acupuncture combined with HCG in the treatment of LUFS. | 60 | Luteinization Syndrome of UNot reporteduptured Follicles |  |
| 134 | Li.F | 2015 | Shenzhen Journal of Integrated Traditional Chinese and Western Medicine | 1 | 1 | none | RCT | To explore the clinical effect of acupuncture and moxibustion in the treatment of anovulatory infertility. | 102 | Ovulation Disorder |  |
| 135 | Li.J.Y | 2015 | Yunnan University of Chinese Medicine | 2 | 1 | none | RCT | To observe the effect of time-phased acupuncture and qi conduction method on ovulatory function in ovulatory disorders of infertility. | 72 | Ovulation Disorder |  |
| 136 | Zhou.C.H | 2015 | Journal of Practical Traditional Chinese Medicine | 1 | 1 | none | RCT | To observe the efficacy of abdominal acupuncture in combination with Dong's acupuncture in the treatment of infertility. | 102 | Not reported |  |
| 137 | Li.S.S | 2015 | Chinese Medicine Modern Distance Education of China | 1 | 1 | none | RCT | To observe the clinical efficacy of acupuncture-assisted metformin on infertility in obese polycystic ovary syndrome. | 150 | Polycystic Ovary Syndrome |  |
| 138 | Sheng.Y.H | 2015 | Asia-Pacific Traditional Medicine | 1 | 1 | none | RCT | To observe the clinical effects of the combination of needle and medicine in the treatment of clomiphene-resistant polycystic ovary syndrome infertility. | 100 | Polycystic Ovary Syndrome |  |
| 139 | Sheng.Y.H;Liu.H.Z;Jiang.C.Y;Deng.L.Q;J.Xu | 2015 | Hebei Journal of Traditional Chinese Medicine | 1 | 1 | none | RCT | Not reported | 138 | Ovulation Disorder |  |
| 140 | Xu.Z.H;Wang.Y.F | 2015 | Women's Health Research | 1 | 1 | none | RCT | To study the clinical effects of acupuncture combined with ovulation promotion in the treatment of infertile women with phlegm-damp polycystic ovary syndrome. | 76 | Polycystic Ovary Syndrome |  |
| 141 | T.Li | 2014 | Heilongjiang University of Chinese Medicine | 2 | 1 | none | RCT | Through the observation of the clinical efficacy of acupuncture combined with clomiphene in the treatment of luteal insufficiency infertility, and compared with the simple clomiphene group and objective evaluation, to actively explore the role and mechanism of acupuncture in the treatment of this disease. it is clear that acupuncture plays an important role in improving ovarian function, promoting follicular development and improving luteal function. To put forward a safe and effective clinical treatment scheme for the treatment of infertility caused by luteal insufficiency. | 36 | Luteal Phase Defect |  |
| 142 | Wu.J.M;Zhuo.Y.Y;Zhong.Y.L;Chen.Q.L | 2014 | Jilin Journal of Chinese Medicine | 1 | 1 | none | RCT | To observe the clinical efficacy of acupuncture with acupuncture points in the treatment of polycystic ovary syndrome (PCOS) infertility. | 60 | Polycystic Ovary Syndrome |  |
| 143 | Liu.Q,L;H.Yin;Wang.J.L;Liu.Y.L;Y.Xie;Wang.S.K | 2014 | Shanghai Journal of Acupuncture and Moxibustion | 1 | 1 | none | RCT | To observe the clinical efficacy of fire acupuncture in the treatment of endometriosis. | 58 | Endometriosis |  |
| 144 | D.Yang | 2014 | Hunan University of Chinese Medicine | 2 | 1 | none | RCT | To observe the efficacy of acupuncture and clomiphene together in inducing ovulation rate and expectation rate in women with polycystic ovary syndrome. | 60 | Polycystic Ovary Syndrome |  |
| 145 | Li.L;H.Mo;B.Wen;J.Zhang;Y.Li;Chen.W.F;Huang.X.W;Peng.X.H;Li.J.M;Li.Z.M | 2014 | China Journal of Traditional Chinese Medicine | 1 | 1 | Provincial and ministerial projects | RCT | To observe the efficacy of acupuncture combined with metformin in the treatment of infertility in obese polycystic ovary syndrome. | 153 | Polycystic Ovary Syndrome |  |
| 146 | Jin.L.H;Zhang.H.B;Li.A.P;Y.Yu | 2014 | Zhejiang Journal of Traditional Chinese Medicine | 1 | 1 | none | RCT | Not reported | 60 | Luteinization Syndrome of UNot reporteduptured Follicles |  |
| 147 | Kang.X.J | 2013 | Heilongjiang University of Chinese Medicine | 2 | 1 | none | RCT | To observe the therapeutic method of acupuncture for soothing the liver and relieving depression, regulating qi and dredging collaterals, regulating Chong Ren, to achieve the effect of ovulation and pregnancy, to observe its clinical effect and explore its mechanism. | 65 | Luteinization Syndrome of UNot reporteduptured Follicles |  |
| 148 | M.Xu;Tian.Y.Z;Zhu.X.J;Hu.X.D;He.T.T | 2013 | Chinese Acupuncture & Moxibustion | 1 | 1 | National project | RCT | To observe the clinical efficacy of plum blossom needle in the treatment of follicular dysplasia. | 50 | Follicular Dysplasia |  |
| 149 | Wang.X.L;Wei.Y.L;L.Yan | 2013 | Shanghai Journal of Acupuncture and Moxibustion | 1 | 1 | none | RCT | Observation of the clinical efficacy of heat-sensitive moxibustion as the main treatment for hyperprolactinemia-induced infertility | 85 | Hyperprolactinemia |  |
| 150 | Ke.D.Y | 2013 | Maternal and Child Health Care of China | 1 | 1 | Provincial and ministerial projects | RCT | To investigate the clinical characteristics of tubal obstructive infertility and analyze the significance of combined laparoscopic postoperative laparoscopic treatment with abdominal acupuncture in improving pregnancy rate and promoting recovery | 82 | Obstruction of Fallopian Tube |  |
| 151 | Gao.J.F | 2013 | Hebei Journal of Traditional Chinese Medicine | 1 | 1 | none | RCT | Not reported | 76 | Not reported |  |
| 152 | Ke.Y.F | 2013 | Fujian University of Traditional Chinese Medicine | 2 | 1 | none | RCT | To study the effects of auricular acupressure on urinary LH peak and ovulation in patients with kidney deficiency and blood addiction type of luteinized uNot reporteduptured follicle syndrome (LUFS), and to explore its mechanism of action, so as to provide a basis and method for clinical treatment. | 56 | Luteinization Syndrome of UNot reporteduptured Follicles |  |
| 153 | Huang.Y.H | 2012 | Guangzhou University of Chinese Medicine | 2 | 1 | none | RCT | To observe the clinical efficacy of acupuncture point burial therapy in the treatment of antisperm immune infertility, and to explore more effective and less toxic treatment methods for the treatment of antisperm immune infertility, so as to provide a basis for the clinical treatment of antisperm immune infertility. | 60 | Endocrine Infertility |  |
| 154 | Cao.Y.M | 2012 | Chinese and Foreign Health Abstract | 1 | 1 | none | RCT | A study of the clinical effectiveness of acupuncture in the treatment of patients suffering from ovulatory infertility. | 78 | Ovulation Disorder |  |
| 155 | B.Zhou;B.Wang | 2012 | Shandong Journal of Traditional Chinese Medicine | 1 | 1 | none | RCT | To observe the effect of acupuncture combined with clomiphene citrate on endometrial receptive state in patients with ovulation induction. | 150 | Not reported |  |
| 156 | Yu, Xue-su; Yan, Xing-qiang; Shen, Yu-yu | 2012 | © Shanghai Research Institute of Acupuncture and Meridian and Springer-Verlag Berlin Heidelberg 2012 | 1 | 2 | none | RCT | To observe the efficacy of acupuncture combined with medicine in the treatment of dysovulatory infertility. | 36 | Ovulation Disorder |  |
| 157 | H.Qu | 2012 | Guangzhou University of Chinese Medicine | 2 | 1 | none | RCT | Through scientific and rigorous clinical experimental research, to observe and evaluate the clinical efficacy of abdominal acupuncture in the treatment of ovulation disturbance in polycystic ovary syndrome of kidney deficiency and blood stasis. | 53 | Polycystic Ovary Syndrome |  |
| 158 | Guo.J.F;J.Liu;P.Shi | 2012 | Chinese Journal of Information on Traditional Chinese Medicine | 1 | 1 | none | RCT | Not reported | 124 | Ovulation Disorder |  |
| 159 | Deng.Y.Z | 2012 | Chinese Acupuncture & Moxibustion | 1 | 1 | none | RCT | Clinical efficacy of acupoint catgut embedding in the treatment of anovulatory infertility | 82 | Not reported |  |
| 160 | He.T.T | 2011 | Guangzhou University of Chinese Medicine | 2 | 1 | none | RCT | To compare the clinical efficacy of plum blossom acupuncture by following the meridian and tapping on the Chong Ren Dou Zhanzhong (the belt of the Dragon) with that of Western medicine, and to explore the possible mechanism of action of plum blossom acupuncture in the treatment of FM, as well as the clinical advantages of plum blossom acupuncture combined with Chinese medicine, to explore a new method that is safe, simple, non-invasive and effective, and to provide a scientific basis for its application and promotion in clinical practice. | 56 | Follicular Dysplasia |  |
| 161 | L.Liu | 2011 | Guangzhou University of Chinese Medicine | 2 | 1 | none | RCT | To observe the safety of abdominal acupuncture in the treatment of LUFS, to evaluate the improvement of ovarian haematology by abdominal targeting and to explore a new safe, non-invasive, effective and comfortable method for the treatment of LUFS. | 50 | Luteinization Syndrome of UNot reporteduptured Follicles |  |
| 162 | Mi.X.L | 2011 | Jiangsu Journal of Traditional Chinese Medicine | 1 | 1 | none | RCT | To observe and analyze the clinical efficacy of electroacupuncture in the treatment of ovulatory dysfunctional infertility. | 48 | Ovulation Disorder |  |
| 163 | Xie,Y.P;X.Wu;X.Lun;L.Liu;Mao.Y.P | 2011 | China Medical Herald | 1 | 1 | Provincial and ministerial projects | RCT | To observe the clinical efficacy of the buried threads at Yu and Shang points on immune infertility and the effect on serum β-endorphin (β-EP). | 120 |  | Immune Infertility |
| 164 | H.Teng;Liu.Y.L;Wang.J.L;Y.Xie | 2011 | Shanghai Journal of Acupuncture and Moxibustion | 1 | 1 | none | RCT | To observe the clinical efficacy of acupuncture plus electroacupuncture in the treatment of infertility. | 65 | Not reported |  |
| 165 | Du.X.Z;Zhao.F.H;Liu,Y.C | 2011 | Chinese Traditional Medicine Information | 1 | 1 | none | RCT | To observe the clinical efficacy of acupuncture plus electroacupuncture in the treatment of infertility. | 132 | Ovulation Disorder |  |
| 166 | Yang.X.Q | 2011 | Chinese Manipulation and Rehabilitation Medicine | 1 | 1 | none | RCT | To observe the clinical effect of acupuncture and moxibustion in the treatment of dysovulatory infertility. | 132 | Ovulation Disorder |  |
| 167 | You.Q.F | 2011 | Journal of Community Medicine | 1 | 1 | none | RCT | To investigate the efficacy of laparoscopy combined with electroacupuncture in the treatment of distal tubal obstructive infertility. | 144 | Obstruction of Fallopian Tube |  |
| 168 | Lan.X.L;H.Feng;Mao.Z.Y;Quan.X.C | 2011 | Clinical Medicine & Engineering | 1 | 1 | Provincial and ministerial projects | RCT | To investigate the effect of post-laparoscopic treatment with abdominal acupuncture on the outcome of tubal obstructive infertility. | 80 | Obstruction of Fallopian Tube |  |
| 169 | Pan.B.Q;L.Chen;Mo.L.L;Wu.L.M | 2011 | Chinese Journal of Medicinal Guide | 1 | 1 | none | RCT | Not reported | 26 | Polycystic Ovary Syndrome |  |
| 170 | L.Xu;B.Xie;D.Xu;P.Ji | 2010 | New Chinese Medicine | 1 | 1 | none | RCT | To observe the clinical efficacy of moxibustion combined with clomiphene (CC) in the treatment of dysovulatory infertility with kidney deficiency. | 90 | Ovulation Disorder |  |
| 171 | Wei.L.X;Zhou.J.P;S.Xu;Shao.X.M;Liang.R.F | 2010 | China Journal of Traditional Chinese Medicine | 1 | 1 | Provincial and ministerial projects | RCT | To observe the clinical efficacy of moxibustion combined with clomiphene (CC) in the treatment of dysovulatory infertility with kidney deficiency. | 179 | Ovulation Disorder |  |
| 172 | Yang.H.W;Huang.X.Y | 2010 | Shanghai Journal of Acupuncture and Moxibustion | 1 | 1 | none | RCT | To observe the clinical efficacy of acupuncture in the treatment of infertility with incomplete luteal function. | 50 | Luteal Phase Defect |  |
| 173 | Dieterle, S; Li, C; Greb, R; Bartzsch, F; Hatzmann, W; Huang, D; Fleckenstein, J | 2010 | Fertility and Sterility | 1 | 2 | none | RCT | Not reported | 52 |  | Idiopathic Male Infertility |
| 174 | Sun.X.L;Zhang.R.Luo.G.G | 2010 | Journal of Traditional Chinese Medicine | 1 | 1 | none | RCT | Not reported | 106 | Endocrine Infertility |  |
| 175 | F.Zhang;Bian.F.X;liu.F. | 2010 | Journal of Chinese Physician | 1 | 1 | none | RCT | Study of the effectiveness of triple therapy in the treatment of insulin-resistant polycystic ovary syndrome (PCOS)-induced infertility | 59 | Polycystic Ovary Syndrome |  |
| 176 | Zhu.X.J | 2009 | Guangzhou University of Chinese Medicine | 2 | 1 | none | RCT | To demonstrate the exact efficacy of Bo's abdominal acupuncture plus infrared irradiation in the treatment of this disease. | 50 | Luteinization Syndrome of UNot reporteduptured Follicles |  |
| 177 | F.Hu | 2009 | Guangzhou University of Chinese Medicine | 2 | 1 | none | RCT | By observing the clinical effect of traditional Chinese medicine moxibustion on endometrium in ovulation induction cycle, we tried to find a way to improve the side effects of clomiphene citrate. | 60 | Ovulation Disorder |  |
| 178 | M.Liu | 2008 | Shandong University of Traditional Chinese Medicine | 2 | 1 | none | RCT | To observe the effect of acupuncture combined with intracavitary physiotherapy on ovulation induction and the level of vascular endothelial growth factor in follicular fluid of patients with luteinized uNot reporteduptured follicular syndrome of kidney deficiency. | 64 | Luteinization Syndrome of UNot reporteduptured Follicles |  |
| 179 | Yu.X.S;Yan.X.Q;Shen.Y.Y | 2008 | Shanghai Journal of Acupuncture and Moxibustion | 1 | 1 | none | RCT | To observe the efficacy of acupuncture combined with clomiphene in the treatment of dysovulatory infertility by ultrasound. | 36 | Ovulation Disorder |  |
| 180 | Song.F.J;Zhen.s.l;Ma.D.Z | 2008 | Chinese Acupuncture & Moxibustion | 1 | 1 | none | RCT | To explore the clinical efficacy and mechanism of acupuncture and moxibustion in the treatment of dysovulatory infertility. | 120 | Ovulation Disorder |  |
| 181 | Zhen.S.L;Song.F.J;Ma.dD.Z | 2007 | Journal of Clinical Acupuncture and Moxibustion | 1 | 1 | Provincial and ministerial projects | RCT | To observe and evaluate the efficacy of acupuncture and moxibustion in the treatment of dysovulatory infertility. | 80 | Ovulation Disorder |  |
| 182 | D.Chen;Chen.S.R;Shi.X.L;Guo.F.L;Zhu.Y,K;S.Li;Cai.M.X;Deng.L.H;H.Xu | 2007 | Chinese Acupuncture & Moxibustion | 1 | 1 | none | RCT | To explore the clinical effect of acupuncture therapy in the treatment of polycystic ovary syndrome. | 121 | Polycystic Ovary Syndrome |  |
| 183 | Yang.J.R;Ma.Y.Y;Liu.Y.L;Wang.H.L;Z.Liu | 2005 | Chinese Acupuncture & Moxibustion | 1 | 1 | Provincial and ministerial projects | RCT | Comparing the differences in the efficacy of acupuncture and ovulation-promoting western medicine in the treatment of endocrine disorders of infertility | 240 | Endocrine Infertility |  |
| 184 | Yang.Y.H;Hong.J.Y;Wei.D.Y;Chen.X.M;J.Lin | 2005 | Journal of Guangdong Medical University | 1 | 1 | none | RCT | To observe the clinical efficacy of acupuncture in the treatment of infertility caused by polycystic ovary syndrome. | 126 | Polycystic Ovary Syndrome |  |
| 185 | Chen.D;Xu.H.G;Hong.Y.B;Chen.S.R;H.Xu;Cai.M.X | 2005 | Chinese Acupuncture & Moxibustion | 1 | 1 | National project | RCT | To observe the clinical effect of needle picking therapy on infertile patients with varicocele after operation. | 122 |  | Varicocele |
| 186 | Zheng.W,G | 2004 | Journal of Gansu University of Chinese Medicine | 1 | 1 | none | RCT | Study on immunosuppressive effect of electroacupuncture on male immune Infertility | 318 |  | Immune Infertility |
| 187 | Xin.L;L.Rong | 2004 | Chinese Acupuncture & Moxibustion | 1 | 1 | School level project | RCT | To observe the therapeutic effect of Yuyuan acupoint matching method on male immune infertility and its effect on antisperm antibody (AsAb). | 100 |  | Immune Infertility |
| 188 | D.Chen;Shi.X.L;Cai.M.X;Guo.F.L;Deng.L.H;H.Xu | 2004 | Chinese Journal of Integrated Traditional and Western Medicine | 1 | 1 | National project | RCT | Not reported | 257 | Ovulation Disorder |  |
| 189 | Li.H.X | 2003 | Shandong University of Traditional Chinese Medicine | 2 | 1 | none | RCT | To observe the efficacy of electroacupuncture in the treatment of luteinized uNot reporteduptured follicle syndrome of kidney deficiency and explore its mechanism. | 60 | Luteinization Syndrome of UNot reporteduptured Follicles |  |
| 190 | D.Chen;Luo.J.X;Cai.M.X;Guo.F.L;Zhang.Y.J | 2003 | Journal of Jinan University | 1 | 1 | none | RCT | To observe the clinical efficacy of acupuncture plus nerve point injection in the treatment of abnormal sperm in patients with infertility. | 189 |  | Immune Infertility |
| 191 | Ma.Y.Y;Yang.J.R | 2003 | Chinese medical theory and practice | 1 | 1 | none | RCT | Not reported | 120 | Endocrine Infertility |  |
| 192 | He.X.Y | 1998 | Shanghai Journal of Acupuncture and Moxibustion | 1 | 1 | none | RCT | Not reported | 108 |  | Idiopathic Male Infertility |

**Continue to table S2**

| ID | Trial group VS Control group Type ^c^ | Trial group acupoint selection schemed ^d^ | Types of acupuncture regimens in the experimental group ^e^ | Frequency of acupuncture treatment ^f^ | Course of treatment^g^ | Adverse effects | Follow-up | Acupuncturist qualificati-on | Protocol Registrati-on | Research hypothesis | Outcom-es^h^ | Results^i^ | Conclusion^j^ |
| --- | --- | --- | --- | --- | --- | --- | --- | --- | --- | --- | --- | --- | --- |
| 1 | 2 | 2 | 4 | 1 | 1 | Nausea, vomiting, dizziness | Not reported | Not reported | Not reported | Not reported | 1 | 1 | 1 |
| 2 | 2 | 1 | 5 | 1 | 1 | Not reported | Not reported | Not reported | Not reported | Not reported | 1 | 1 | 1 |
| 3 | 2 | 2 | 5 | 1 | 1 | Not reported | Not reported | Not reported | China Clinical Trial Center (Registration number: ChiCTR-IOR-15007358) | Not reported | 1 | 1 | 1 |
| 4 | 1 | 1 | 5 | 1 | 2 | Not reported | Not reported | Not reported | Not reported | Not reported | 1 | 1 | 1 |
| 5 | 2 | 1 | 5 | 1 | 1 | Not reported | Not reported | Not reported | Not reported | Not reported | 1 | 1 | 1 |
| 6 | 1 | 1 | 5 | 1 | 2 | Not reported | Not reported | Not reported | Not reported | Not reported | 1 | 1 | 1 |
| 7 | 2 | 1 | 1 | 1 | 1 | Not reported | Not reported | Not reported | Not reported | Not reported | 1 | 1 | 1 |
| 8 | 2 | 3 | 5 | Not report | Not report | Not reported | Not reported | Not reported | Not reported | Not reported | 1 | 1 | 1 |
| 9 | Acupuncture + lifestyle intervention + Western medicine VS lifestyle intervention + Western medicine | 1 | 5 | Once every 10 days | 1 | Not reported | 1 year | Not reported | Not reported | Not reported | 1 | 1 | 1 |
| 10 | 2 | 1 | 5 | 1 | 1 | Not reported | Not reported | Not reported | Not reported | Not reported | 1 | 1 | 1 |
| 11 | 2 | 1 | 5 | 1 | 1 | Not reported | 1 year | Not reported | Not reported | Not reported | 1 | 1 | 1 |
| 12 | Acupuncture + core muscle rehabilitation exercise + Western medicine VS Western medicine | 1 | 5 | Once every two weeks | 1 | Not reported | Not reported | Not reported | Not reported | Not reported | 1 | 1 | 1 |
| 13 | 2 | 1 | 5 | Once every two weeks | 1 | Not reported | Not reported | Not reported | Not reported | Not reported | 1 | 1 | 1 |
| 14 | 2 | 1 | 1 | 1 | 1 | Not reported | 1 menstrual cycle | Not reported | Not reported | Not reported | 1 | 1 | 1 |
| 15 | 1 | 1 | 5 | 1 | 1 | Not reported | Not reported | Not reported | Not reported | Not reported | 1 | 1 | 1 |
| 16 | 1 | 1 | 5 | 1 | 1 | Not reported | Not reported | Not reported | Not reported | Not reported | 1 | 1 | 1 |
| 17 | 1 | 1 | 5 | 1 | 20 days | Not reported | Not reported | Not reported | Not reported | Not reported | 1 | 1 | 1 |
| 18 | 2 | 1 | 5 | 1 | 1 | Adverse reactions were reported, but no specific adverse reactions were reported | Not reported | Not reported | Not reported | Not reported | 1 | 1 | 1 |
| 19 | 2 | 1 | 5 | 2 | 1 | Not reported | 1-6 months | Not reported | Not reported | Not reported | 1 | 1 | 1 |
| 20 | 1 | 1 | 5 | 1 | 1 | Not reported | 3个月 | Not reported | Not reported | Not reported | 1 | 1 | 2 |
| 21 | Acupuncture VS comfort acupuncture | 1 | 5 | 2 | 1 | Not reported | Not reported | Not reported | Not reported | Not reported | 1 | 1 | 2 |
| 22 | 1 | 1 | 5 | 1 | 2 | Not reported | Not reported | Not reported | Not reported | Not reported | 1 | 1 | 1 |
| 23 | 2 | 1 | 5 | Once a week | 1 | Adverse reactions were reported, but no specific adverse reactions were reported | 1-6 months | Not reported | Not reported | Not reported | 1 | 1 | 1 |
| 24 | 2 | 1 | 5 | 1 | Not reported | Not reported | Not reported | Not reported | Not reported | Not reported | 1 | 1 | 1 |
| 25 | 1 | 1 | 5 | 2 | 1 | Not reported | 1-9 months | Not reported | Not reported | Not reported | 1 | 1 | 1 |
| 26 | 2 | 1 | 5 | 2 | 1 month | Not reported | Not reported | Not reported | Not reported | Not reported | 1 | 1 | 1 |
| 27 | 2 | 1 | 5 | 1 | 2 | Not reported | 1-6 months | Not reported | Not reported | Not reported | 1 | 1 | 1 |
| 28 | 2 | 1 | 5 | 1 | 1 | Not reported | 1 year | Not reported | Not reported | Not reported | 1 | 1 | 1 |
| 29 | 1 | 1 | 5 | 1 | 1 | Adverse reactions were reported, but no specific adverse reactions were reported | Not reported | Not reported | Not reported | Not reported | 1 | 1 | 2 |
| 30 | 2 | 1 | 5 | Not reported | 1 month | Adverse reactions were reported, but no specific adverse reactions were reported | Not reported | Not reported | Not reported | Not reported | 1 | 1 | 1 |
| 31 | 2 | 1 | 5 | Once every two weeks | 1 | Not reported | Not reported | Not reported | Not reported | Not reported | 1 | 1 | 1 |
| 32 | 2 | 1 | 5 | 1 | Not reported | Adverse reactions were reported, but no specific adverse reactions were reported | Not reported | Not reported | Not reported | Not reported | 1 | 1 | 1 |
| 33 | 2 | 1 | 5 | 1 | 1 | Not reported | Not reported | Not reported | Not reported | Not reported | 1 | 1 | 1 |
| 34 | 1 | 1 | 5 | 1 | 1 month | Not reported | Follow-up after 1 year | Not reported | Not reported | Undefined | 1 | 1 | 1 |
| 35 | 2 | 1 | 5 | Not reported | 1 | Not reported | Follow-up visit not reported | Not reported | Not reported | Undefined | 1 | 1 | 1 |
| 36 | 1 | 1 | 5 | 1 | 1 | Not reported | Follow-up visit not reported | Not reported | Not reported | Undefined | 1 | 1 | 1 |
| 37 | 2 | 1 | 5 | Not reported | 1 | Not reported | Follow-up visit not reported | Not reported | Not reported | Undefined | 1 | 1 | 1 |
| 38 | 2 | 1 | 5 | 1 | 1 | Not reported | Follow-up visit not reported | Not reported | Not reported | Undefined | 1 | 1 | 1 |
| 39 | 2 | 1 | 5 | 2 | 1 | Not reported | Follow-up visit not reported | Not reported | Not reported | Undefined | 1 | 1 | 1 |
| 40 | 1 | 1 | 5 | 1 | 1 | Not reported | Follow-up after 6 months | Not reported | Not reported | Undefined | 1 | 1 | 1 |
| 41 | 2 | 1 | 5 | 2 treatments per week | 1 | 1 participant experienced dizziness and nausea; three participants experienced abnormal lower limb soreness | Follow-up visit not reported | Not reported | Not reported | Undefined | 1 | 1 | 1 |
| 42 | 2 | 1 | 5 | 1 treatment every 2 weeks | 1 | Abdominal distension and nausea after treatment in 3 cases, mild lower abdominal pain in 2 cases, subcutaneous hemorrhage after needling in 2 cases, one experienced hair loss, and 1 case of hypoglycemia | Follow-up visit not reported | Not reported | Not reported | Undefined | 1 | 1 | 2 |
| 43 | 2 | 1 | 5 | 2 | 1 | 1 participant had a small bruise on his abdomen after treatment | Follow-up visit not reported | Not reported | Not reported | Undefined | 3 | 2 | 1 |
| 44 | acupuncture + surgery VS surgery | 1 | 5 | 1 | 1 | Three participants experienced nausea and vomiting after treatment, and four participants experienced rash | Follow-up after 1 year | Not reported | Not reported | Undefined | 1 | 1 | 1 |
| 45 | HCG combined with BO's abdominal acupuncture VS HCG VS acupuncture | 1 | 3 | 1 | 1 | Not reported | Follow-up visit not reported | Not reported | Not reported | Undefined | 1 | 1 | 1 |
| 46 | 2 | 1 | 5 | 1 | 1 | Not reported | Follow-up visit not reported | Not reported | Not reported | Undefined | 1 | 1 | 1 |
| 47 | 2 | 1 | 5 | 1 | 1 | Not reported | Follow-up after 6 months | Not reported | Not reported | Undefined | 1 | 1 | 1 |
| 48 | 1 | 1 | 5 | 1 | 1 | Not reported | Follow-up after 1 year | Not reported | Not reported | Undefined | 1 | 1 | 1 |
| 49 | 2 | 1 | 5 | 1 | 2 months | Not reported | Follow-up visit not reported | Not reported | Not reported | Undefined | 1 | 1 | 1 |
| 50 | acupuncture VS western medicine VS condom isolation | 2 | 5 | 2 | 1 | Not reported | Follow-up visit not reported | Not reported | Not reported | Undefined | 1 | 1 | 1 |
| 51 | 1 | 1 | 5 | 1 | 1 | Not reported | Follow-up visit not reported | Not reported | Not reported | Undefined | 3 | 1 | 1 |
| 52 | 2 | 1 | 5 | 1 | 1 | Not reported | Follow-up visit not reported | Not reported | Not reported | Undefined | 1 | 1 | 1 |
| 53 | 2 | 1 | 5 | 2 | Not reported | Not reported | Follow-up visit not reported | Not reported | Not reported | Undefined | 1 | 1 | 1 |
| 54 | 2 | 2 | 5 | 2 | 1 | Not reported | Follow-up after 6 months | Not reported | Not reported | Undefined | 1 | 1 | 1 |
| 55 | 2 | 1 | 5 | 1 | 2 months | Not reported | Follow-up visit not reported | Not reported | Not reported | Undefined | 3 | 1 | 1 |
| 56 | 1 | 1 | 5 | 1 | 1 | Not reported | Follow-up after 3 months | Not reported | Not reported | Undefined | 1 | 2 | 2 |
| 57 | 2 | 1 | 5 | 1 | 1 | No acupuncture-related adverse events | Follow-up visit not reported | Not reported | Not reported | Undefined | 1 | 1 | 1 |
| 58 | 1 | 1 | 5 | Not reported | Not reported | Not reported | Follow-up visit not reported | Not reported | Not reported | Undefined | 1 | 1 | 1 |
| 59 | 2 | 1 | 5 | 1 | 9 menstrual cycles | Not reported | Follow-up visit not reported | Not reported | Not reported | Undefined | 1 | 1 | 1 |
| 60 | 2 | 1 | 5 | 1 | 1 | Not reported | Follow-up after 6 months | Not reported | Not reported | Undefined | 1 | 1 | 1 |
| 61 | Metformin VS acupuncture VS Metformin combined with acupuncture | 1 | 5 | 1 | 1 | Not reported | Follow-up after 6 months | Not reported | Not reported | Undefined | 1 | 1 | 1 |
| 62 | 2 | 1 | 5 | Not reported | 2 | No acupuncture-related adverse events | Follow-up after 1 year | Not reported | Not reported | Undefined | 1 | 1 | 1 |
| 63 | 2 | 1 | 2 | 1 | 1 | Not reported | Follow-up visit not reported | Not reported | Not reported | Undefined | 1 | 1 | 1 |
| 64 | 2 | 2 | 5 | 1 | 1 | Not reported | Follow-up visit not reported | Not reported | Not reported | Undefined | 1 | 1 | 1 |
| 65 | 1 | 1 | 5 | Not reported | 45-75days | Not reported | Follow-up visit not reported | Not reported | Not reported | Undefined | 1 | 1 | 1 |
| 66 | 2 | 1 | 5 | 1 | 1 | Not reported | Follow-up visit not reported | Not reported | Not reported | Undefined | 1 | 2 | 2 |
| 67 | 2 | 1 | 5 | 1 | 1 | No acupuncture-related adverse events | Follow-up after 6 months | Not reported | Not reported | Undefined | 1 | 1 | 1 |
| 68 | 2 | 1 | 5 | 1 | 1 | Not reported | Follow-up visit not reported | Not reported | Not reported | Undefined | 1,2 | 2 | 2 |
| 69 | 2 | 1 | 5 | 2 | 1 | Not reported | No specific follow-up time was reported | Not reported | Not reported | Undefined | 1 | 3 | 1 |
| 70 | 1 | 1 | 1 | 1 | Not reported | Not reported | Follow-up visit not reported | Not reported | Not reported | Undefined | 1 | 2 | 1 |
| 71 | 2 | 2 | 5 | 1 | 1 | Not reported | Follow-up visit not reported | Not reported | Not reported | Undefined | 1 | 1 | 1 |
| 72 | 2 | 1 | 5 | 1 treatment every 10 days | 1 | Loss of appetite after treatment in 1 participant | Follow-up visit not reported | Not reported | Not reported | Undefined | 1 | 1 | 1 |
| 73 | 1 | 1 | 5 | 1 | Not reported | Not reported | Follow-up visit not reported | Not reported | Not reported | Undefined | 1 | 1 | 1 |
| 74 | 2 | 2 | 5 | Not reported | 1 | Not reported | Follow-up visit not reported | Not reported | Not reported | Undefined | 1 | 1 | 1 |
| 75 | moxibustion group VS the placebo moxibustion group | 1 | 5 | 1 | 1 | 1 participant allergic to tape | Follow-up after 2 weeks | Not reported | Not reported | Undefined | 1,3 | 1 | 1 |
| 76 | 2 | 1 | 5 | 2 | 1 | Not reported | Follow-up after 3 months | Not reported | Not reported | Undefined | 1 | 3 | 1 |
| 77 | 2 | 1 | 5 | 1 | 2 months | Not reported | Follow-up visit not reported | Not reported | Not reported | Undefined | 1 | 1 | 1 |
| 78 | 1 | 1 | 5 | 1 | 1 | Not reported | Follow-up visit not reported | Not reported | Not reported | Undefined | 1 | 2 | 2 |
| 79 | 1 | 1 | 5 | 1 | 1 | Not reported | Follow-up visit not reported | Not reported | Not reported | Undefined | 1 | 3 | 1 |
| 80 | 2 | 2 | 3 | 1 | 1 | Not reported | Follow-up visit not reported | Not reported | Not reported | Undefined | 1 | 1 | 1 |
| 81 | 1 | 2 | 5 | 1 | 1 | Not reported | Follow-up visit not reported | Not reported | Not reported | Undefined | 1 | 2 | 2 |
| 82 | 2 | 1 | 5 | 1 | 1month | No acupuncture-related adverse events | Follow-up visit not reported | Not reported | Not reported | Effectiveness study | 1 | 1 | 1 |
| 83 | 2 | 1 | 5 | 1 | 2 | Nausea after treatment in 1 case, fainting during acupuncture in 2 cases, back pain in 2 cases | Follow-up visit not reported | Not reported | Not reported | Undefined | 1 | 1 | 1 |
| 84 | 1 | 2 | 3 | 1 | Not reported | Not reported | Follow-up visit not reported | Not reported | Not reported | Undefined | 1 | 1 | 1 |
| 85 | 2 | 1 | 5 | 1 | 1 | Not reported | Follow-up visit not reported | Not reported | Not reported | Undefined | 1 | 1 | 1 |
| 86 | 2 | 2 | 5 | 1 | 1 | Not reported | Follow-up after 3 months | Not reported | Not reported | Undefined | 1 | 2 | 2 |
| 87 | 2 | 2 | 5 | 1 | 1 | Not reported | Follow-up after 3 months | Not reported | Not reported | Undefined | 1 | 3 | 1 |
| 88 | 2 | 2 | 5 | 2 | 2 | Not reported | Follow-up visit not reported | Not reported | Not reported | Undefined | 1 | 1 | 1 |
| 89 | acupuncture + surgery VS surgery | 2 | 5 | 1 | 1 | Not reported | Follow-up visit not reported | Not reported | Not reported | Undefined | 1 | 1 | 1 |
| 90 | 2 | 1 | 5 | 1 | 1 | Subcutaneous hemorrhage after treatment in 3 participants | Follow-up after 3 months | Not reported | Not reported | Undefined | 1 | 2 | 2 |
| 91 | 2 | 1 | 5 | 1 | 9 menstrual cycles | Not reported | Follow-up visit not reported | Not reported | Not reported | Undefined | 1 | 3 | 1 |
| 92 | 2 | 1 | 5 | 1 | 9 menstrual cycles | Not reported | Follow-up visit not reported | Not reported | Not reported | Undefined | 1 | 3 | 1 |
| 93 | 2 | 2 | 5 | 1 | 1 | No acupuncture-related adverse events | Follow-up visit not reported | Not reported | Not reported | Undefined | 1 | 1 | 1 |
| 94 | 1 | 1 | 3 | 1 | 1 | Not reported | Follow-up visit not reported | Not reported | Not reported | Undefined | 1 | 1 | 1 |
| 95 | Thunder—Fire moxibustion combined ovulation  Monitoring VS ovulation monitoring | 1 | 5 | 1 | 2 | Not reported | Follow-up visit not reported | Not reported | Not reported | Undefined | 1 | 1 | 1 |
| 96 | Prednisolone Acetate Tablets VS acupuncture VS Prednisolone Acetate Tablets combined with acupuncture | 1 | 5 | 1 | 1 month | Not reported | Follow-up after 6 months | Not reported | Not reported | Undefined | 1 | 2 | 2 |
| 97 | 2 | 2 | 5 | 1 | 1 | Not reported | Follow-up visit not reported | Not reported | Not reported | Undefined | 1 | 1 | 1 |
| 98 | 2 | 1 | 3 | 1 | Not reported | Not reported | Follow-up visit not reported | Not reported | Not reported | Undefined | 1 | 1 | 1 |
| 99 | 1 | 1 | 5 | Not reported | 1 | Not reported | Follow-up visit not reported | Not reported | Not reported | Undefined | 1 | 3 | 1 |
| 100 | 2 | 1 | 5 | 1 | 1 | Not reported | Follow-up visit not reported | Not reported | Not reported | Undefined | 1 | 1 | 1 |
| 101 | 1 | 1 | 5 | 1 | 1 | Not reported | Follow-up visit not reported | Not reported | Not reported | Undefined | 1 | 1 | 1 |
| 102 | 2 | 1 | 5 | 1 | Not reported | Not reported | Follow-up visit not reported | Not reported | Not reported | Undefined | 1 | 1 | 1 |
| 103 | 2 | 2 | 5 | 1 | 4 months | Not reported | Follow-up visit not reported | Not reported | Not reported | Undefined | 1 | 1 | 1 |
| 104 | 1 | 1 | 5 | 1 | 1 | Not reported | Follow-up visit not reported | Not reported | Not reported | Undefined | 1 | 2 | 1 |
| 105 | 1 | 2 | 5 | 1 | Not reported | Not reported | Follow-up visit not reported | Not reported | Not reported | Undefined | 1 | 1 | 1 |
| 106 | 2 | 1 | 5 | 1 | 1 | Not reported | Follow-up visit not reported | Not reported | Not reported | Undefined | 1 | 1 | 1 |
| 107 | 2 | 1 | 5 | 1 treatment per week | 1 | Subcutaneous hemorrhage or swelling sensation occurred at the acupoint of catgut embedding in a few patients | Follow-up visit not reported | Not reported | Not reported | Undefined | 1 | 1 | 1 |
| 108 | 2 | 2 | 5 | 1 | 1 | Not reported | Follow-up visit not reported | Not reported | Not reported | Undefined | 1 | 1 | 1 |
| 109 | 2 | 1 | 5 | 1 | Not reported | Not reported | Follow-up visit not reported | Not reported | Not reported | Undefined | 1 | 1 | 1 |
| 110 | 1 | 2 | 5 | 1 | 1 | Not reported | Follow-up visit not reported | Not reported | Not reported | Undefined | 1 | 1 | 1 |
| 111 | 2 | 2 | 5 | 1 | 1 | Not reported | Follow-up visit not reported | Not reported | Not reported | Undefined | 1 | 2 | 1 |
| 112 | 2 | 1 | 5 | 1 | Not reported | Not reported | Follow-up after 3 months | Not reported | Not reported | Undefined | 1 | 1 | 1 |
| 113 | 1 | 1 | 5 | 1 | 1 | Not reported | Follow-up visit not reported | Not reported | Not reported | Undefined | 1 | 1 | 1 |
| 114 | 1 | 1 | 5 | 1 | 2 months | Nausea and vomiting after treatment in 1 participant | Follow-up visit not reported | Not reported | Not reported | Undefined | 1 | 1 | 1 |
| 115 | 1 | 3 | 5 | 1 | 1 | Fainting during acupuncture after treatment in 1 case, stuck needles in 2 cases and 2 experienced stomach upset | Follow-up after 1 year | Not reported | Not reported | Undefined | 1 | 1 | 1 |
| 116 | acupuncture+operation VS operation | 1 | 5 | 1 | 1 | not explicit | No specific follow-up time was reported. | Not reported | Not reported | undefined | 1 | 1 | 1 |
| 117 | acupuncture+operation VS operation | 1 | 5 | 1 | 1 | not explicit | 1 year | Not reported | Not reported | undefined | 1 | 1 | 1 |
| 118 | 1 | 1 | 5 | 1 | 1 | not explicit | Not reported | Not reported | Not reported | undefined | 1 | 1 | 1 |
| 119 | 2 | 1 | 1 | 1 | 6 months | not explicit | Not reported | Not reported | Not reported | undefined | 1 | 1 | 1 |
| 120 | 2 | 1 | 5 | 1 | 1 | not explicit | Not reported | Not reported | Not reported | undefined | 1 | 1 | 1 |
| 121 | 1 | 2 | 5 | 2 | 2 | not explicit | No specific follow-up time was reported. | Not reported | Not reported | undefined | 1 | 2 | 2 |
| 122 | acupuncture VS sham acupuncture | 1 | 5 | 2 | 6 weeks | not explicit | Not reported | Not reported | Not reported | undefined | 3 | 3 | 2 |
| 123 | 1 | 1 | 5 | 1 | 1 | not explicit | Not reported | Not reported | Not reported | undefined | 1 | 1 | 1 |
| 124 | acupuncture+behavior therapy+western medicine VS western medicine | 1 | 5 | 1 | Not reported | not explicit | Not reported | Not reported | Not reported | undefined | 1 | 1 | 1 |
| 125 | acupuncture+infrared radiation VS blank | 1 | 3 | 1 | a menstrual cycle | not explicit | Not reported | Not reported | Not reported | undefined | 1 | 1 | 1 |
| 126 | 2 | 1 | 5 | 1 | 2 | not explicit | Not reported | Not reported | Not reported | undefined | 1 | 2 | 2 |
| 127 | acupuncture+western medicine+intracavitary physiotherapy VS western medicine | 1 | 5 | 1 | 1 | not explicit | Not reported | Not reported | Not reported | undefined | 1 | 1 | 1 |
| 128 | 2 | 1 | 5 | 1 | Not reported | not explicit | Not reported | Not reported | Not reported | undefined | 1 | 1 | 1 |
| 129 | 1 | 1 | 5 | 1 | 1 | not explicit | 6 months | Not reported | Not reported | undefined | 1 | 3 | 1 |
| 130 | 1 | 1 | 5 | Not reported | 1 | not explicit | 6 months | Not reported | Not reported | undefined | 1 | 1 | 1 |
| 131 | 1 | 1 | 5 | 1 | Not reported | not explicit | Not reported | Not reported | Not reported | undefined | 1 | 1 | 1 |
| 132 | 1 | 1 | 5 | Once a week | 1 | not explicit | one year | Not reported | Not reported | undefined | 1 | 1 | 1 |
| 133 | 1 | 1 | 5 | 1 | 1 | not explicit | 43 months | Not reported | Not reported | undefined | 1 | 1 | 1 |
| 134 | 1 | 1 | 1 | 1 | 4 months | not explicit | Not reported | Not reported | Not reported | undefined | 1 | 1 | 1 |
| 135 | 1 | 2 | 5 | 1 | 1 | not explicit | 1 year | Not reported | Not reported | undefined | 1 | 2 | 2 |
| 136 | 2 | 1 | 3 | 1 | 1 | not explicit | Not reported | Not reported | Not reported | undefined | 1 | 1 | 1 |
| 137 | 2 | 1 | 5 | Once a week | 9 weeks | not explicit | Not reported | Not reported | Not reported | undefined | 3 | 1 | 1 |
| 138 | 1 | 2 | 5 | Once a month | 20 months | not explicit | Not reported | Not reported | Not reported | undefined | 1 | 1 | 1 |
| 139 | 2 | 1 | 3 | 1 | 1 | not explicit | Not reported | Not reported | Not reported | undefined | 1；3 | 1 | 1 |
| 140 | acupuncture+operation VS operation | 1 | 5 | 1 | 1 | not explicit | No specific follow-up time was reported. | Not reported | Not reported | undefined | 1 | 1 | 1 |
| 141 | acupuncture+operation VS operation | 1 | 5 | 1 | 1 | not explicit | 1 year | Not reported | Not reported | undefined | 1 | 1 | 1 |
| 142 | 1 | 1 | 5 | 1 | 1 | not explicit | Not reported | Not reported | Not reported | undefined | 1 | 1 | 1 |
| 143 | 2 | 1 | 1 | 1 | 6 months | not explicit | Not reported | Not reported | Not reported | undefined | 1 | 1 | 1 |
| 144 | 2 | 1 | 5 | 1 | 1 | not explicit | Not reported | Not reported | Not reported | undefined | 1 | 1 | 1 |
| 145 | 1 | 2 | 5 | 2 | 2 | not explicit | No specific follow-up time was reported. | Not reported | Not reported | undefined | 1 | 2 | 2 |
| 146 | acupuncture VS sham acupuncture | 1 | 5 | 2 | 6 weeks | not explicit | Not reported | Not reported | Not reported | undefined | 3 | 3 | 2 |
| 147 | 1 | 1 | 5 | 1 | 1 | not explicit | Not reported | Not reported | Not reported | undefined | 1 | 1 | 1 |
| 148 | acupuncture+behavior therapy+western medicine VS western medicine | 1 | 5 | 1 | Not reported | not explicit | Not reported | Not reported | Not reported | undefined | 1 | 1 | 1 |
| 149 | acupuncture+infrared radiation VS blank | 1 | 3 | 1 | a menstrual cycle | not explicit | Not reported | Not reported | Not reported | undefined | 1 | 1 | 1 |
| 150 | 2 | 1 | 5 | 1 | 2 | not explicit | Not reported | Not reported | Not reported | undefined | 1 | 2 | 2 |
| 151 | acupuncture+western medicine+intracavitary physiotherapy VS western medicine | 1 | 5 | 1 | 1 | not explicit | Not reported | Not reported | Not reported | undefined | 1 | 1 | 1 |
| 152 | 2 | 1 | 5 | 1 | Not reported | not explicit | Not reported | Not reported | Not reported | undefined | 1 | 1 | 1 |
| 153 | 1 | 1 | 5 | 1 | 1 | not explicit | 6 months | Not reported | Not reported | undefined | 1 | 3 | 1 |
| 154 | 1 | 1 | 5 | Not reported | 1 | not explicit | 6 months | Not reported | Not reported | undefined | 1 | 1 | 1 |
| 155 | 1 | 2 | 5 | 1 | 1 | not explicit | 2 years | Not reported | Not reported | undefined | 1 | 1 | 1 |
| 156 | 1 | 2 | 5 | 1 | 1 | not explicit | Not reported | Not reported | Not reported | undefined | 1 | 1 | 1 |
| 157 | 1 | 1 | 5 | 1 | Not reported | not explicit | Not reported | Not reported | Not reported | undefined | 1 | 1 | 1 |
| 158 | 1 | 1 | 5 | 1 | 1 | not explicit | 43 months | Not reported | Not reported | undefined | 1 | 1 | 1 |
| 159 | 1 | 1 | 1 | 1 | 4 months | not explicit | Not reported | Not reported | Not reported | undefined | 1 | 1 | 1 |
| 160 | 1 | 2 | 5 | 1 | 1 | not explicit | 1 year | Not reported | Not reported | undefined | 1 | 2 | 2 |
| 161 | 2 | 1 | 3 | 1 | 1 | not explicit | Not reported | Not reported | Not reported | undefined | 1 | 1 | 1 |
| 162 | 2 | 1 | 5 | Once a week | 9 weeks | not explicit | Not reported | Not reported | Not reported | undefined | 3 | 1 | 1 |
| 163 | 1 | 2 | 5 | Once a month | 20 months | not explicit | Not reported | Not reported | Not reported | undefined | 1 | 1 | 1 |
| 164 | 2 | 1 | 3 | 1 | 1 | not explicit | Not reported | Not reported | Not reported | undefined | 1；3 | 1 | 1 |
| 165 | acupuncture+operation VS operation | 1 | 5 | 1 | 1 | not explicit | No specific follow-up time was reported. | Not reported | Not reported | undefined | 1 | 1 | 1 |
| 166 | acupuncture+operation VS operation | 1 | 5 | 1 | 1 | not explicit | 1 year | Not reported | Not reported | undefined | 1 | 1 | 1 |
| 167 | 1 | 1 | 5 | 1 | 1 | not explicit | Not reported | Not reported | Not reported | undefined | 1 | 1 | 1 |
| 168 | 2 | 1 | 1 | 1 | 6 months | not explicit | Not reported | Not reported | Not reported | undefined | 1 | 1 | 1 |
| 169 | 2 | 1 | 5 | 1 | 1 | not explicit | Not reported | Not reported | Not reported | undefined | 1 | 1 | 1 |
| 170 | 1 | 2 | 5 | 2 | 2 | not explicit | No specific follow-up time was reported. | Not reported | Not reported | undefined | 1 | 2 | 2 |
| 171 | acupuncture VS sham acupuncture | 1 | 5 | 2 | 6 weeks | not explicit | Not reported | Not reported | Not reported | undefined | 3 | 3 | 2 |
| 172 | 1 | 1 | 5 | 1 | 1 | not explicit | Not reported | Not reported | Not reported | undefined | 1 | 1 | 1 |
| 173 | acupuncture+behavior therapy+western medicine VS western medicine | 1 | 5 | 1 | Not reported | not explicit | Not reported | Yes | Not reported | undefined | 1 | 1 | 1 |
| 174 | acupuncture+infrared radiation VS blank | 1 | 3 | 1 | a menstrual cycle | not explicit | Not reported | Not reported | Not reported | undefined | 1 | 1 | 1 |
| 175 | 2 | 1 | 5 | 1 | 2 | not explicit | Not reported | Not reported | Not reported | undefined | 1 | 2 | 2 |
| 176 | acupuncture+western medicine+intracavitary physiotherapy VS western medicine | 1 | 5 | 1 | 1 | not explicit | Not reported | Not reported | Not reported | undefined | 1 | 1 | 1 |
| 177 | 2 | 1 | 5 | 1 | Not reported | not explicit | Not reported | Not reported | Not reported | undefined | 1 | 1 | 1 |
| 178 | 1 | 1 | 5 | 1 | 1 | not explicit | 6 months | Not reported | Not reported | undefined | 1 | 3 | 1 |
| 179 | 1 | 1 | 5 | Not reported | 1 | not explicit | 6 months | Not reported | Not reported | undefined | 1 | 1 | 1 |
| 180 | 1 | 2 | 5 | 1 | 1 | not explicit | 2 years | Not reported | Not reported | undefined | 1 | 1 | 1 |
| 181 | 1 | 2 | 5 | 1 | 1 | not explicit | Not reported | Not reported | Not reported | undefined | 1 | 1 | 1 |
| 182 | 1 | 1 | 5 | 1 | Not reported | not explicit | Not reported | Not reported | Not reported | undefined | 1 | 1 | 1 |
| 183 | 1 | 1 | 5 | Once a week | 1 | not explicit | one year | Not reported | Not reported | undefined | 1 | 1 | 1 |
| 184 | 1 | 1 | 5 | 1 | 1 | not explicit | 43 months | Not reported | Not reported | undefined | 1 | 1 | 1 |
| 185 | 1 | 1 | 1 | 1 | 4 months | not explicit | Not reported | Not reported | Not reported | undefined | 1 | 1 | 1 |
| 186 | 1 | 2 | 5 | 1 | 1 | not explicit | 1 year | Not reported | Not reported | undefined | 1 | 2 | 2 |
| 187 | 2 | 1 | 3 | 1 | 1 | not explicit | Not reported | Not reported | Not reported | undefined | 1 | 1 | 1 |
| 188 | 2 | 1 | 5 | Once a week | 9 weeks | not explicit | Not reported | Not reported | Not reported | undefined | 3 | 1 | 1 |
| 189 | 1 | 2 | 5 | Once a month | 20 months | not explicit | Not reported | Not reported | Not reported | undefined | 1 | 1 | 1 |
| 190 | 2 | 1 | 3 | 1 | 1 | not explicit | Not reported | Not reported | Not reported | undefined | 1；3 | 1 | 1 |
| 191 | acupuncture+operation VS operation | 1 | 5 | 1 | 1 | not explicit | No specific follow-up time was reported. | Not reported | Not reported | undefined | 1 | 1 | 1 |
| 192 | acupuncture+operation VS operation | 1 | 5 | 1 | 1 | not explicit | 1 year | Not reported | Not reported | undefined | 1 | 1 | 1 |

Notes:a：1-Journal article；2-Thesis；3-Conference paper

b：1-Chinese；2-English

c：1-Acupuncture VS Western medicine；2- Acupuncture + Western medicine VS Western medicine；other types can be directly translated

d：1-Fixed acupoints；2- Fixed acupoints combined with syndrome differentiation；3- Individualized acupoint selection

e：1-Acupuncture teaching materials；2-Clinical Guidelines for Acupuncture and Moxibustion；3-Senior TCM Expert Experience；4-personal experience；5-Undefined

f：1-3 times a week or more；2-2 times a week；the rest can be directly translated

g：1-3 months or 3 menstrual cycle；2-6 months or 6 menstrual cycle；the rest can be directly translated

h：1- Pregnancy rate；2-Live Birth Rates；3- Semen motility, quantity, fragmentation

i：1-Positive results；2-Negative results；3-Not reported

j：1- the curative effect of the acupuncture group is better than that of the control group；2- the curative effect of the acupuncture and moxibustion group is comparable to the control group

**Table S3： Characteristics of 73 case series**

| ID | Author(same study ID, use -1; -2) | Year of publication | Journal/Conference Name/Dissertation Awarding Unit | Type of publication^a^ | language^b^ | Fund support | Type of Study | Research purposes | sample size | Diseases or causes of female infertility | Diseases or causes of male infertility |
| --- | --- | --- | --- | --- | --- | --- | --- | --- | --- | --- | --- |
| 1 | Yin.Y.Q | 2019 | China association of acupuncture and moxibustion | 3 | 1 | Provincial project | Case series | To explore the effect of acupuncture for menstruation regulation and pregnancy promotion on ovarian function in patients with premature ovarian insufficiency and the factors that affect pregnancy in patients with this disease. | 86 | Premature ovarian insufficiency |  |
| 2 | Duan.L.N | 2019 | Chinese acupuncture and moxibustion | 1 | 1 | Not reported | Case series | NOT REPORTED | 30 | Immune infertility |  |
| 3 | Wang.Z.Y | 2018 | World Latest Medicine Information | 1 | 1 | Not reported | Case series | Analysis of the clinical effect of acupuncture on male infertility. | 140 |  | idiopathic infertility |
| 4 | Liu.Y.L | 2016 | China's Naturopathy | 1 | 1 | Not reported | Case series | Not reported | 21 | Not reported |  |
| 5 | Fang.J.X | 2016 | China Medical Devices Information | 1 | 1 | Not reported | Case series | To observe the curative effect of acupuncture on infertility with less sperm. | 34 |  | idiopathic infertility |
| 6 | Chen.Z.Y | 2014 | Chinese acupuncture and moxibustion | 1 | 1 | Not reported | Case series | Not reported | 42 | not reported in details |  |
| 7 | Liu.L.W | 2013 | Chinese Medicine Modern Distance Education of China | 1 | 1 | Not reported | Case series | Not reported | 50 | Not reported |  |
| 8 | Y.Xu | 2013 | World Journal of Acupuncture-Moxibustion | 1 | 2 | Not reported | Case series | To explore the clinical effect of acupuncture and moxibustion to induce ovulation. | 40 | Ovulation disorders |  |
| 9 | Wang.Q.Y | 2012 | Heilongjiang University of Traditional Chinese Medicine | 2 | 1 | Not reported | Case series | To observe the clinical curative effect of acupuncture on infertility patients with kidney deficiency type luteal insufficiency and its effect on serum estrogen and progesterone | 16 | luteal insufficiency |  |
| 10 | Huang.H.T | 2011 | Shanghai Journal of Acupuncture and Moxibustion | 1 | 1 | Not reported | Case series | Not reported | 13 | Not reported |  |
| 11 | R.Zhao | 2011 | Shanghai Journal of Acupuncture and Moxibustion | 1 | 1 | Not reported | Case series | Not reported | 20 | Polycystic ovarian syndrome |  |
| 12 | Yin.D.H | 2011 | Hainan Medical Journal | 1 | 1 | Provincial project | Case series | It is planned to conduct a clinical study on electroacupuncture in the treatment of ovulatory infertility patients, and to preliminarily explore its mechanism of action. | 40 | Ovulation disorders |  |
| 13 | Wang.Q.M | 2010 | Chinese acupuncture and moxibustion | 1 | 1 | Not reported | Case series | Not reported | 32 |  | varicocele |
| 14 | Ji.C.Y | 2009 | Shandong University of Traditional Chinese Medicine | 2 | 1 | Not reported | Case series | To conduct clinical and experimental research on electroacupuncture intervention in the treatment of ovulatory infertility (kidney-yang deficiency type), and to explore its mechanism of action. | 30 | Ovulation disorders |  |
| 15 | Tang.H.X | 2009 | Yunnan Journal of Traditional Chinese Medicine and Materia Medica | 1 | 1 | Not reported | Case series | Not reported | 85 | luteal insufficiency |  |
| 16 | Huang.T.M | 2009 | Clinical Medicine & Engineering | 1 | 1 | Not reported | Case series | Not reported | 12 |  | Not reported |
| 17 | Song.G.H | 2009 | Chinese acupuncture and moxibustion | 1 | 1 | Not reported | Case series | Not reported | 80 |  | idiopathic infertility |
| 18 | M.Xu | 2008 | Chinese Medicine Modern Distance Education of China | 1 | 1 | Not reported | Case series | To observe the curative effect of pulse-cutting acupuncture on ovulatory infertility. | 70 | Ovulation disorders |  |
| 19 | S.Wang | 2008 | China's Naturopathy | 1 | 1 | Not reported | Case series | Not reported | 34 | Ovulation disorders |  |
| 20 | Liu.H.X | 2007 | Journal of Chinese Modern Traditional Chinese Medicine | 1 | 1 | Not reported | Case series | To observe the curative effect of Ziwuliuzhu acupuncture on infertility. | 58 | Not reported |  |
| 21 | Song.S.H | 2007 | Shaanxi Journal of Traditional Chinese Medicine | 1 | 1 | Not reported | Case series | To observe the clinical efficacy of "burning mountain fire" acupuncture manipulation in the treatment of infertility due to deficiency of kidney yang. | 50 | Not reported |  |
| 22 | Peng.M.H | 2005 | Journal of Acupuncture and Tuina Science | 1 | 2 | Not reported | Case series | Not reported | 39 |  | idiopathic infertility |
| 23 | Wang.C.N | 2005 | Chinese acupuncture and moxibustion | 1 | 1 | Not reported | Case series | Not reported | 17 | Not reported |  |
| 24 | li.J.X | 2005 | Shenzhen Journal of Integrated Traditional Chinese and Western Medicine | 1 | 1 | Not reported | Case series | Not reported | 17 | Not reported |  |
| 25 | Kuang.S.H | 2005 | Journal of Clinical Medicine And Pharmacy | 1 | 1 | Not reported | Case series | Not reported | 38 | tubal obstrucrion |  |
| 26 | X.Lun | 2005 | Journal of Acupuncture and Tuina Science | 1 | 2 | Colonel-level project | Case series | To observe the curative effect of stagnant acupuncture on immune infertility. | 79 |  | immune infertility |
| 27 | Yang.X.Z | 2005 | The Journal of Medical Theory and Practice | 1 | 1 | Not reported | Case series | Not reported | 58 | tubal obstrucrion |  |
| 28 | Pang.B.Z-1 | 2004 | Jilin Journal of Traditional Chinese Medicine | 1 | 1 | Not reported | Case series | Not reported | 106 | not reported in details |  |
| 29 | Pang.B.Z-2 | 2004 | Heilongjiang Journal of Traditional Chinese Medicine | 1 | 1 | Not reported | Case series | Discuss the effect of acupuncture on spermatogenesis. | 128 |  | idiopathic infertility |
| 30 | Chen.X.Y | 2004 | Chinese acupuncture and moxibustion | 1 | 1 | Not reported | Case series | Not reported | 38 |  | chronic epididymitis |
| 31 | Lv.J.X | 2004 | LIAONING JOURNAL OF TRADITIONAL CHINESE MEDICINE | 1 | 1 | Not reported | Case series | Not reported | 628 |  | idiopathic infertility |
| 32 | F.Chen | 2003 | Jilin Journal of Traditional Chinese Medicine | 1 | 1 | Not reported | Case series | Not reported | 79 |  | idiopathic infertility |
| 33 | Sha.G.E | 2002 | Hebei Journal of Traditional Chinese Medicine | 1 | 1 | Not reported | Case series | Not reported | 78 | not reported in details |  |
| 34 | Wang.C.Y | 2002 | ChineseJournalofClinicalRehabilitation | 1 | 1 | Not reported | Case series | Not reported | 62 | not reported in details |  |
| 35 | Mou.Q.R | 2001 | Hunan Journal of Traditional Chinese Medicine | 1 | 1 | Not reported | Case series | Not reported | 36 | tubal obstrucrion |  |
| 36 | Liu.C.W | 2001 | Chinese acupuncture and moxibustion | 1 | 1 | Not reported | Case series | Not reported | 12 | Not reported |  |
| 37 | Luo.D.P | 2001 | Henan Traditional Chinese Medicine | 1 | 1 | Not reported | Case series | Not reported | 11 | luteinized uNot reporteduptured follicle syndrome |  |
| 38 | Wang.S.Z | 2001 | Western Journal of Traditional Chinese Medicine | 1 | 1 | Not reported | Case series | Not reported | 13 |  | Not reported |
| 39 | Yang.Z.Q | 2000 | Jiangxi journal of traditional chinese medicine | 1 | 1 | Not reported | Case series | Not reported | 48 |  | urinary tract infection |
| 40 | Shi.Z.H | 2000 | Chinese acupuncture and moxibustion | 1 | 1 | Not reported | Case series | Not reported | 30 | ovulatory dysfunction |  |
| 41 | Yu.Z.B | 2000 | Chinese acupuncture and moxibustion | 1 | 1 | Not reported | Case series | Not reported | 34 |  | idiopathic infertility |
| 42 | Li.X.N | 1999 | Journal of clinical acupuncture and moxibustion | 1 | 1 | Not reported | Case series | Not reported | 28 | Not reported |  |
| 43 | Xu.S.Q | 1999 | Journal of clinical acupuncture and moxibustion | 1 | 1 | Not reported | Case series | Not reported | 48 | Not reported |  |
| 44 | Piao.Z.T | 1999 | China association of acupuncture and moxibustion | 1 | 1 | Not reported | Case series | Not reported | 13 | tubal obstrucrion |  |
| 45 | Lu.R.C | 1998 | Shaanxi Journal of Traditional Chinese Medicine | 1 | 1 | Not reported | Case series | Not reported | 118 | Not reported |  |
| 46 | Chang.J.L | 1998 | Shanghai Journal of Acupuncture and Moxibustion | 1 | 1 | Not reported | Case series | Not reported | 32 | Not reported |  |
| 47 | Jin.P.L | 1998 | Journal of practical traditional chinese medicine | 1 | 1 | Not reported | Case series | Not reported | 30 | tubal obstrucrion |  |
| 48 | Ding.H.J | 1998 | Journal of clinical acupuncture and moxibustion | 1 | 1 | Not reported | Case series | Not reported | 31 | tubal obstrucrion |  |
| 49 | Q.Chen | 1998 | Hebei Journal of Traditional Chinese Medicine | 1 | 1 | Not reported | Case series | Not reported | 66 | Not reported |  |
| 50 | Lian.Y.L | 1998 | Journal of clinical acupuncture and moxibustion | 1 | 1 | Not reported | Case series | Not reported | 83 |  | idiopathic infertility |
| 51 | Kou.J.M | 1997 | Henan Traditional Chinese Medicine | 1 | 1 | Not reported | Case series | Not reported | 50 | Not reported |  |
| 52 | Siterman, S | 1997 | Archives of Andrology | 1 | 2 | Not reported | Case series | Not reported | 16 |  | idiopathic infertility |
| 53 | P.Chen | 1997 | Modern Diagnosis & Treatment | 1 | 1 | Not reported | Case series | Not reported | 189 |  | idiopathic infertility |
| 54 | Qi.L.L | 1996 | Chinese Journal of Ultrasonography | 1 | 1 | Not reported | Case series | Not reported | 21 | luteinized uNot reporteduptured follicle syndrome |  |
| 55 | D.Bai | 1996 | Chinese acupuncture and moxibustion | 1 | 1 | Not reported | Case series | Not reported | 66 |  | idiopathic infertility |
| 56 | He.X.Y | 1996 | Chengdu pharmaceutical | 1 | 1 | Not reported | Case series | Not reported | 35 |  | idiopathic infertility |
| 57 | Pang.G.Q | 1995 | Chinese Manipulation & Rehabilitation Medicine | 1 | 1 | Not reported | Case series | Not reported | 10 | not reported in details |  |
| 58 | L.Luo | 1995 | Shanghai Journal of Acupuncture and Moxibustion | 1 | 1 | Not reported | Case series | Not reported | 36 |  | idiopathic infertility |
| 59 | Qian.Z.Y | 1995 | Journal of clinical acupuncture and moxibustion | 1 | 1 | Not reported | Case series | Not reported | 54 |  | idiopathic infertility |
| 60 | Yu.G.P | 1995 | Journal of clinical acupuncture and moxibustion | 1 | 1 | Not reported | Case series | Not reported | 86 |  | idiopathic infertility |
| 61 | Shi.Z.D | 1994 | Shanghai Journal of Acupuncture and Moxibustion | 1 | 1 | Not reported | Case series | Not reported | 30 | Not reported |  |
| 62 | Jie.Y.X | 1994 | Shandong Medical Journal | 1 | 1 | Not reported | Case series | Not reported | 68 | Not reported |  |
| 63 | Ji.Y.H | 1994 | Journal of Sichuan of Traditional Chinese Medicine | 1 | 1 | Not reported | Case series | Not reported | 30 |  | Not reported |
| 64 | Liu.Y.J | 1993 | Jilin Journal of Traditional Chinese Medicine | 1 | 1 | Not reported | Case series | Not reported | 41 | ovulatory dysfunction |  |
| 65 | Liu.M.X | 1992 | Hunan Journal of Traditional Chinese Medicine | 1 | 1 | Not reported | Case series | Not reported | 137 | Not reported |  |
| 66 | He.X.L | 1992 | Shanxi Journal of Traditional Chinese Medicine | 1 | 1 | Not reported | Case series | Not reported | 46 |  | not ejaculating |
| 67 | Li.W.T | 1990 | Shaanxi Journal of Traditional Chinese Medicine | 1 | 1 | Not reported | Case series | Not reported | 53 | posterior uterus |  |
| 68 | J.Liu | 1989 | Jiangsu Journal of Traditional Chinese Medicine | 1 | 1 | Not reported | Case series | Not reported | 78 |  | Not reported |
| 69 | Jiang.Z.Q | 1988 | Guangxi journal of traditional chinese medicine | 1 | 1 | Not reported | Case series | Not reported | 11 | Not reported |  |
| 70 | Chen.Y.X | 1988 | Tianjin traditional Chinese medicine | 1 | 1 | Not reported | Case series | Not reported | 45 |  | Not reported |
| 71 | Yang.Z.Y | 1986 | Jiangxi journal of traditional chinese medicine | 1 | 1 | Not reported | Case series | Not reported | 15 | ovulatory dysfunction |  |
| 72 | Tao.Z.X | 1985 | Journal of acupuncture and moxibustion | 1 | 1 | Not reported | Case series | Not reported | 16 |  | Not reported |
| 73 | Yang.Y.H | 1984 | Journal of Nanjing Railway College | 1 | 1 | Not reported | Case series | Not reported | 28 |  | Not reported |

**Continue to table S3**

| ID | Trial group acupoint selection scheme^d^ | Types of acupuncture regimens in the experimental group ^e^ | Frequency of acupuncture treatment^f^ | Course of treatment^g^ | Adverse effects | Follow-up | Acupuncturist qualification | Protocol Registration | Outcomes ^h^ |
| --- | --- | --- | --- | --- | --- | --- | --- | --- | --- |
| 1 | 1 | 3 | 1 | 1 | Needle stagnation, subcutaneous congestion | Not reported | Not reported | Not reported | 1 |
| 2 | 1 | 5 | 1 time a week | 2 months | Not reported | 6 months | Not reported | Not reported | 1 |
| 3 | 1 | 5 | Not reported | Not reported | Not reported | Not reported | Not reported | Not reported | 1；3 |
| 4 | 1 | 5 | 1 | 4 months | Not reported | Not reported | Not reported | Not reported | 1 |
| 5 | 1 | 5 | 1 | Not reported | Not reported | Not reported | Not reported | Not reported | 3 |
| 6 | 2 | 5 | 1 | 1 menstrual cycle | Not reported | Not reported | Not reported | Not reported | 1 |
| 7 | 1 | 5 | 1 | Unclear | Not reported | Not reported | Not reported | Not reported | 1 |
| 8 | 1 | 5 | 1 | 1 | Not reported | Follow-up was reported, but no specific follow-up time was reported | Not reported | Not reported | 1 |
| 9 | 2 | 5 | 1 | 1 | Not reported | 3 months | Not reported | Not reported | 1 |
| 10 | 1 | 5 | 1 | 1 | Not reported | Not reported | Not reported | Not reported | 1 |
| 11 | 2 | 5 | 2-3 times a week | Unclear | Not reported | 2 years | Not reported | Not reported | 1 |
| 12 | 1 | 5 | 1 | 1 | Not reported | 1 years | Not reported | Not reported | 1 |
| 13 | 2 | 5 | 1 | 6 weeks | Not reported | 1 years | Not reported | Not reported | 1；3 |
| 14 | 1 | 5 | 1 | 1 | Not reported | Not reported | Not reported | Not reported | 1 |
| 15 | 1 | 5 | 1 | 1 | Not reported | Not reported | Not reported | Not reported | 1 |
| 16 | 1 | 4 | Not reported | Unclear | Not reported | Not reported | Not reported | Not reported | 1 |
| 17 | 1 | 4 | 1 | Unclear | Not reported | Not reported | Not reported | Not reported | 3 |
| 18 | 1 | 5 | 1 | 2 weeks | Not reported | Not reported | Not reported | Not reported | 1 |
| 19 | 2 | 5 | 1 | Unclear | Not reported | Not reported | Not reported | Not reported | 1 |
| 20 | 3 | 5 | Not reported | Unclear | Not reported | Not reported | Not reported | Not reported | 1 |
| 21 | 1 | 1 | 1 time a week | Unclear | Not reported | Not reported | Not reported | Not reported | 1 |
| 22 | 1 | 5 | 1 | 1 | Not reported | Not reported | Not reported | Not reported | 1 |
| 23 | 1 | 5 | 1 | Not reported | Not reported | Not reported | Not reported | Not reported | 1 |
| 24 | 3 | 5 | 1-2 times a week | Unclear | Not reported | Not reported | Not reported | Not reported | 1 |
| 25 | 3 | 5 | 1 | Unclear | Not reported | Not reported | Not reported | Not reported | 1 |
| 26 | 1 | 5 | 1 | 2 months | Not reported | Not reported | Not reported | Not reported | 3 |
| 27 | 1 | 5 | 1 | 20 days | Not reported | Not reported | Not reported | Not reported | 1 |
| 28 | 2 | 5 | 1 | 1 menstrual cycle | Not reported | Not reported | Not reported | Not reported | 1 |
| 29 | 2 | 5 | 1 | 5 months | Not reported | Not reported | Not reported | Not reported | 1 |
| 30 | 1 | 5 | 1 | Unclear | Not reported | Not reported | Not reported | Not reported | 1 |
| 31 | 2 | 5 | 1 time a week | Not reported | Not reported | Follow-up was reported, but no specific follow-up time was reported | Not reported | Not reported | 1 |
| 32 | 1 | 5 | 1 | Not reported | Not reported | Not reported | Not reported | Not reported | 1 |
| 33 | 2 | 5 | 1 | Not reported | Not reported | 2 years | Not reported | Not reported | 1 |
| 34 | 2 | 5 | 1 | Not reported | Not reported | 3 years | Not reported | Not reported | 1 |
| 35 | 2 | 5 | 1 | Not reported | Not reported | Not reported | Not reported | Not reported | 1 |
| 36 | 2 | 5 | 1 | Unclear | Not reported | Not reported | Not reported | Not reported | 1 |
| 37 | 1 | 5 | Not reported | Not reported | feel sick and vomit | Not reported | Not reported | Not reported | 1 |
| 38 | 2 | 5 | 1 | Not reported | Not reported | Not reported | Not reported | Not reported | 3 |
| 39 | 2 | 5 | 1 | Not reported | Not reported | Not reported | Not reported | Not reported | 1 |
| 40 | 1 | 5 | 1 | Not reported | Not reported | Not reported | Not reported | Not reported | 1 |
| 41 | 1 | 5 | 1 | Not reported | Not reported | Not reported | Not reported | Not reported | 1；3 |
| 42 | 2 | 5 | 1 | Not reported | Not reported | Not reported | Not reported | Not reported | 1 |
| 43 | 1 | 5 | 1 | Unclear | Not reported | Not reported | Not reported | Not reported | 1 |
| 44 | 2 | 5 | 1 | Unclear | Not reported | Not reported | Not reported | Not reported | 1 |
| 45 | 1 | 5 | once a month | Not reported | Not reported | Not reported | Not reported | Not reported | 1 |
| 46 | 2 | 5 | 1 | Not reported | Not reported | Not reported | Not reported | Not reported | 1 |
| 47 | 2 | 5 | 1 | Not reported | Not reported | Not reported | Not reported | Not reported | 1 |
| 48 | 1 | 5 | Not reported | Unclear | Not reported | Not reported | Not reported | Not reported | 1 |
| 49 | 2 | 5 | 1 | Unclear | Not reported | 2 years | Not reported | Not reported | 1 |
| 50 | 2 | 5 | 1 | Not reported | Not reported | Not reported | Not reported | Not reported | 1；3 |
| 51 | 2 | 5 | 1 | Unclear | Not reported | Not reported | Not reported | Not reported | 1 |
| 52 | 1 | 5 | 2 times a month | 5 weeks | Not reported | Not reported | Not reported | Not reported | 3 |
| 53 | 2 | 5 | 1 | Unclear | Not reported | Not reported | Not reported | Not reported | 1；3 |
| 54 | 1 | 5 | Not reported | Unclear | Not reported | Not reported | Not reported | Not reported | 1 |
| 55 | 1 | 5 | once every two weeks | Unclear | Not reported | Not reported | Not reported | Not reported | 3 |
| 56 | 2 | 5 | 1 | Not reported | Not reported | Not reported | Not reported | Not reported | 1；3 |
| 57 | 1 | 5 | 1 | Not reported | Not reported | Not reported | Not reported | Not reported | 1 |
| 58 | 1 | 5 | Not reported | Not reported | Not reported | Not reported | Not reported | Not reported | 3 |
| 59 | 1 | 5 | Not reported | Unclear | Not reported | Not reported | Not reported | Not reported | 1；3 |
| 60 | 2 | 5 | 1 | Unclear | Not reported | Not reported | Not reported | Not reported | 1；3 |
| 61 | 2 | 5 | 1 | 2 | Not reported | Not reported | Not reported | Not reported | 1 |
| 62 | 2 | 5 | 1 | Unclear | Not reported | Not reported | Not reported | Not reported | 1 |
| 63 | 1 | 5 | 1 | Unclear | Not reported | Not reported | Not reported | Not reported | 1；3 |
| 64 | 2 | 5 | 1 | 2 | Not reported | Not reported | Not reported | Not reported | 1 |
| 65 | 2 | 5 | 1 | Unclear | Not reported | Not reported | Not reported | Not reported | 1 |
| 66 | 1 | 5 | 1 | Unclear | Not reported | Not reported | Not reported | Not reported | 1 |
| 67 | 1 | 5 | 1 | Unclear | Not reported | Not reported | Not reported | Not reported | 1 |
| 68 | 2 | 5 | 1 | Not reported | Not reported | Not reported | Not reported | Not reported | 1；3 |
| 69 | 1 | 5 | 1 | Not reported | Not reported | Not reported | Not reported | Not reported | 1 |
| 70 | 2 | 5 | 1 | Unclear | Not reported | Not reported | Not reported | Not reported | 3 |
| 71 | 1 | 4 | 1 | Unclear | Not reported | Not reported | Not reported | Not reported | 1 |
| 72 | 1 | 5 | 1 | Not reported | Not reported | Follow-up was reported, but no specific follow-up time was reported | Not reported | Not reported | 1；3 |
| 73 | 3 | 5 | Not reported | Not reported | Not reported | Not reported | Not reported | Not reported | 1；3 |

Notes:a：1-Journal article；2-Thesis；3-Conference paper

b：1-Chinese；2-English

d：1-Fixed acupoints；2-Fixed acupoints combined with syndrome differentiation；3-Individualized acupoint selection

e：1-Acupuncture teaching materials；2-Clinical Guidelines for Acupuncture and Moxibustion；3-Senior TCM Expert Experience；4-personal experience；5-Undefined

f：1-3 times a week or more；2-2 times a week；the rest can be directly translated

g：1-3 months or 3 menstrual cycle；2-6 months or 6 menstrual cycle；the rest can be directly translated

h：1-pregnancy rate；2-live birth rate；3-Semen motility, quantity, fragmentation

**Table S4： Characteristics of other 8 studies**

| ID | Author (same study ID, use -1; -2) | Year of publication | Journal/Conference Name/Dissertation Awarding Unit | Type of publication ^a^ | language^b^ | Fund support | Type of Study | Research purposes | sample size | Diseases or causes of female infertility |
| --- | --- | --- | --- | --- | --- | --- | --- | --- | --- | --- |
| 1 | ZHANG Dianrun；LIU Hui；ZENG Linghong；WANG Juhua；WANG Ying | 2023 | Journal of Molecular Imaging | 1 | 1 | National project | Cohort study | The development of follicles in infertile patients with polycystic ovary syndrome was dynamically monitored by transvaginal ultrasonography, and the clinical value of combined acupuncture and drug therapy was discussed to provide further guidance for clinical treatment. | 44 | Polycystic ovary syndrome |
| 2 | Xue Lifeng; Miao Yuxin | 2022 | New traditional Chinese medicine | 1 | 1 | Not reported | CCT | To observe the clinical effect of warm acupuncture and moxibustion combined with clomiphene on infertility of polycystic ovary syndrome (PCOS) and its influence on sex hormones and ovulation. | 80 | Polycystic ovary syndrome |
| 3 | Zheng Yongxia; Yu Jingwei; McWeitchart | 2022 | Journal of practical Chinese medicine | 1 | 1 | Bureau level project | CCT | To explore the clinical effect of warm acupuncture combined with letrozole in the treatment of sterility of polycystic ovary syndrome (PCOS) with kidney deficiency and blood stasis | 100 | Polycystic ovary syndrome |
| 4 | Y.Ye | 2021 | Chinese Journal of Birth Health & Heredity | 1 | 1 | Not reported | CCT（non-randomized current controlled trials） | To observe the effects of yin-yang balance acupuncture, clomiphene citrate tablets, letrozole, three ovulation induction treatment methods on the outcome of assisted pregnancy in patients with ovulation disorders, and provide reference for the treatment of patients with ovulation disorders. | 200 | Ovulation disorders |
| 5 | Z.Dou | 2021 | Acupuncture Research | 1 | 1 | National project | cohort study | To observe the effect of acupuncture at eight points around the navel on the metabolic level and reproductive function of patients with infertility caused by obese polycystic ovary syndrome (PCOS). | 96 | Polycystic Ovary Syndrome |
| 6 | Zhen.S.Z | 2018 | Chinese Acupuncture & Moxibustion | 1 | 1 | Provincial project | cohort study | To observe the clinical efficacy and mechanism of "Tongyuan acupuncture" in the treatment of clomiphene citrate (CC) ovulation cycle infertility patients with ovulatory disorders | 36 | Ovulation disorders |
| 7 | Cai.L.H | 2014 | Advances in Reproductive Sciences | 1 | 2 | Provincial project | CCT（non-randomized current controlled trials） | To evaluate the efficacy of adjunctive acupuncture and moxibustion in the treatment of unexplained infertility. | 80 | Unexplained infertility |
| 8 | Gerhard, I | 1992 | Gynecological Endocrinology | 1 | 2 | National project | Matched control study | Evaluating ear acupuncture for infertility | 90 | not reported in details |

**Continue to table S4**

| ID | Trial group VS control group Type ^c^ | Acupoint selection scheme^d^ | Types of acupuncture regimens in the experimental group ^e^ | Frequency of acupuncture treatment ^f^ | Course of treatment^g^ | Adverse effects | Follow-up | Acupunc-turist qualifica-tion | Protocol Registra-tion | Outcom-es ^h^ | Result ^i^ | Conclusion ^j^ |
| --- | --- | --- | --- | --- | --- | --- | --- | --- | --- | --- | --- | --- |
| 1 | 2 | 1 | 5 | 1 | 1 | Not reported | Not reported | Not reported | Not reported | 1 | 1 | 1 |
| 2 | 2 | 2 | 5 | 1 | 1 | Not reported | Not reported |  |  | 1 | 1 | 1 |
| 3 | 2 | 2 | 5 | 1 | 1 | Not reported | 1-6 months |  |  | 1 | 1 | 1 |
| 4 | Western Medicine A + Acupuncture VS Western Medicine A + Western Medicine B | 2 | 3 | 2-3 times a week | Not reported | Not reported | Not reported | Not reported | Not reported | 1 | 2 | 2 |
| 5 | 1 | 1 | 3 | 1 | 1 | Not reported | Not reported | Not reported | Not reported | 1 | 1 | 1 |
| 6 | 1 | 1 | 3 | Not reported | 2 menstrual cycles | No acupuncture-related adverse events | Not reported | Not reported | Not reported | 1 | 1 | 1 |
| 7 | Acupuncture + Hysterosalpingography VS Hysterosalpingography | 1 | Not reported | 1 | Not reported | Not reported | Not reported | Not reported | Not reported | 1 | 1 | 1 |
| 8 | 1 | 1 | Not reported | 1 time a week | 1 | 2 participants experienced ovarian hyperstimulation after treatment, 2 experienced weight gain, and 1 experienced visual impairment | Not reported | Not reported | Not reported | 1 | 2 | 2 |

Notes:a：1-Journal article；2-Thesis；3-Conference paper

b：1-Chinese；2-English

c：1-acupuncture VS western medicine；2-acupuncture + western medicine VS western medicine；other types can be directly translated

d：1-Fixed acupoints；2-Semi-fixed acupoints；3-Individualized acupoint selection

e：1-Acupuncture teaching materials；2-Clinical Guidelines for Acupuncture and Moxibustion；3-Senior TCM Expert Experience；4-personal experience；5-Undefined

f：1-3 times a week or more；2-2 times a week；the rest can be directly translated

g：1-3 months or 3 menstrual cycle；2-6 months or 6 menstrual cycle；the rest can be directly translated

h：1-pregnancy rate；2-live birth rate；3-Semen motility, quantity, fragmentation

i：1 - positive result；2 - negative result

j：1- the curative effect of the acupuncture group is better than that of the control group；2- the curative effect of the acupuncture and moxibustion group is comparable to the control group

**Table S5 Basic characteristics of the included secondary studies**

| Included Studies | Publication Language | Type of original study included | Participant | Number of studies/sample size | Intervention measure | | Outcome |
| --- | --- | --- | --- | --- | --- | --- | --- |
|  |  |  |  |  | Treatment group | Control group |  |
| Lijie Yang 2023 | English | RCT | Infertility due to PCOS | 6/1410 | Clomiphene + Acupuncture ;  Acupuncture alone | Clomiphene;  sham Acupuncture; placebo | Pregnancy Rates |
| Xin Chen 2022 | English | RCT | Infertility due to PCOS | 9/1159 | Acupuncture combined with Metformin | Metformin | Pregnancy Rates |
| Haiqian Lu 2020 | Chinese | RCT | Infertility due to PCOS | 34/3399 | Acupuncture combined with Ovulation stimulating drugs | Ovulation stimulating drugs | Pregnancy Rates |
| Ranran Gao 2020 | English | RCT | Anovulatory Infertility | 9/1441 | Acupuncture;  Acupuncture combined with Clomiphene | Clomiphene | Pregnancy Rates |
| Yuqing Tan 2019 | Chinese | RCT/q-RCT | Ovulation dysfunction Infertility | 5/442 | Acupuncture | Clomiphene | Pregnancy Rates |
| Chi Eung Danforn Lim 2019 | English | RCT | Infertility due to PCOS | 8/1546 | Acupuncture | sham acupuncture; Relaxation; Clomiphene; Physical Exercise; No Intervention; Diane-35 | Pregnancy Rates; Live birth Rates |
| Xiao Ai 2017 | Chinese | RCT | Ovulation dysfunction Infertility | 12/941 | Acupuncture | Clomiphene; HCG | Pregnancy Rates |

**Continue to Table S5**

| Included Studies | Number of studies/sample size | Effect size | | *I*^2^ | *P* | Conclusion |
| --- | --- | --- | --- | --- | --- | --- |
| Lijie Yang 2023 | 273 cases were studied with Acupuncture;  178 cases were studied with Clomiphene;  464 cases were studied with Acupuncture and Clomiphene | Acupuncture VS Clomiphene | MD = -0.80, 95% CI(-1.84, 0.23) | / | / | Acupuncture is effective in improving the ovulation promoting effect and pregnancy outcome of PCOS patients. The ovulation promoting effect of Acupuncture or combined with Clomiphene is similar to that of Clomiphene alone, but Acupuncture combined with Clomiphene has more advantages in improving the pregnancy rate of PCOS, and it also can reduce the adverse reactions of Clomiphene alone. Acupuncture can be used as a recommended treatment for PCOS. |
|  |  | Acupuncture VS Acupuncture + Clomiphene | MD = 0.29, 95% CI (-0.73, 1.30) | / | / |  |
|  |  | Acupuncture + Clomiphene VS Clomiphene | MD = 1.09, 95% CI (0.39, 1.79) | / | / |  |
| Xin Chen 2022 | 6/756 | RR = 1.35, 95% CI(1.13, 1.63) | | 42% | 0.001 | Based on the results of this study, compared with metformin alone, acupuncture combined with metformin has a positive effect on pregnancy rate, ovulation rate, and insulin resistance in PCOS. However, due to the limitations regarding the number and quality of the included studies, the above conclusions need to be verified by further high-quality studies. |
| Haiqian Lu 2020 | 20/2009 | RR = 1.74, 95%CI( 1.54, 1.97) | | 0% | / | Acupuncture combined with ovulation stimulating drugs can improve the pregnancy outcome of PCOS patients and has a good curative effect. However, due to the small sample size and low risk bias evaluation score of some literatures, the influence of potential publication bias and information bias cannot be excluded. |
| Ranran Gao 2020 | 2/558 | Acupuncture + Clomiphene VS Clomiphene | OR = 1.18, 95% CI (0.83, 1.69) | 4% | 0.36 | Based on the above pooled results of the studies, the use of acupuncture as a monotherapy significantly improved the rate of pregnancy among the study participants compared with the use of Clomiphene alone. However, any results drawn from these studies should be interpreted with caution when considering the context of clinical practice. |
|  | 8/883 | Acupuncture VS Clomiphene | OR = 2.34, 95% CI(1.76, 3.10) | 0% | ﹤0.001 |  |
| Yuqing Tan 2019 | 5/442 | RR = 1.45, 95%CI(1.25, 1.69) | | 0% | ﹤0.001 | Compared with Western medicine, acupuncture can effectively improve the pregnancy rate, promote the rate of periodic ovulation, reduce the rate of abortion, promote the development of follicles and increase the level of E2 hormone. |
| Chi Eung Danforn Lim 2019 | 1/926 | Acupuncture VS Sham Acupuncture | Multiple pregnancy rate: RR = 0.89, 95%CI(0.33, 2.45) | / | 0.83 | For true acupuncture versus sham acupuncture we cannot exclude clinically relevant differences in live birth rate, multiple pregnancy rate, ovulation rate, clinical pregnancy rate or miscarriage.  There are only a limited number of RCTs in this area, limiting our ability to determine effectiveness of acupuncture for PCOS. |
|  |  |  | Live birth rate: RR = 0.97, 95%CI(0.76, 1.24) | / | 0.83 |  |
|  | 3/1117 | Acupuncture VS Sham Acupuncture | Clinical pregnancy rate: RR = 1.03, 95% CI(0.82, 1.29) | 0% | 0.82 |  |
| Xiao Ai 2017 | 11/941 | Acupuncture VS Western Medicine | OR = 2.56, 95%CI (1.94, 3.38) | 0% | ﹤0.001 | The curative effect of acupuncture on ovulation disorder infertility is better than that of western medicine. |

Note: PCOS: Polycystic Ovary Syndrome; HCG: Human Chorionic Gonadotropin
